# Genetic predisposition and antipsychotic treatment effect on metabolic syndrome in schizophrenia: a ten-year follow-up study using the Estonian Biobank

**DOI:** 10.1016/j.lanepe.2024.100914

**Published:** 2024-04-26

**Authors:** Maris Alver, Silva Kasela, Liina Haring, Laura Birgit Luitva, Estonian Biobank Research Team, Health Informatics Research Team, Krista Fischer, Märt Möls, Lili Milani

**Affiliations:** a Estonian Genome Centre, Institute of Genomics, University of Tartu, Riia 23b, Tartu, 51010, Estonia; b Department of Psychiatry, Institute of Clinical Medicine, University of Tartu, Raja 31, Tartu, 50417, Estonia; c Psychiatry Clinic of Tartu University Hospital, Raja 31, Tartu, 50417, Estonia; d Institute of Computer Science, University of Tartu, Narva mnt 18, Tartu, 51009, Estonia; e Institute of Mathematics and Statistics, University of Tartu, Narva mnt 18, Tartu, 51009, Estonia

**Keywords:** Schizophrenia, Metabolic syndrome, Antipsychotics, Treatment, Real world data, Genetics, Polygenic risk scores, Body mass index

## Abstract

**Background:**

Schizophrenia (SCZ) patients exhibit 30% higher prevalence of metabolic syndrome (MetS) compared to the general population with its suboptimal management contributing to increased mortality. Large-scale studies providing real-world evidence of the underlying causes remain limited.

**Methods:**

To address this gap, we used real-world health data from the Estonian Biobank, spanning a median follow-up of ten years, to investigate the impact of genetic predisposition and antipsychotic treatment on the development of MetS in SCZ patients. Specifically, we set out to characterize antipsychotic treatment patterns, genetic predisposition of MetS traits, MetS prognosis, and body mass index (BMI) trajectories, comparing SCZ cases (n = 677) to age- and sex-matched controls (n = 2708).

**Findings:**

SCZ cases exhibited higher genetic predisposition to SCZ (OR = 1.75, 95% CI 1.58–1.94), but lower polygenic burden for increased BMI (OR = 0.88, 95% CI 0.88–0.96) and C-reactive protein (OR = 0.88, 95% CI 0.81–0.97) compared to controls. While SCZ cases showed worse prognosis of MetS (HR 1.95, 95% CI 1.54–2.46), higher antipsychotic adherence within the first treatment year was associated with reduced long-term MetS incidence. Linear mixed modelling, incorporating multiple BMI timepoints, underscored the significant contribution of both, antipsychotic medication, and genetic predisposition to higher BMI, driving the substantially upward trajectory of BMI in SCZ cases.

**Interpretation:**

These findings contribute to refining clinical risk prediction and prevention strategies for MetS among SCZ patients and emphasize the significance of incorporating genetic information, long-term patient tracking, and employing diverse perspectives when analyzing real-world health data.

**Funding:**

EU Horizon 2020, 10.13039/501100004359Swedish Research Council, 10.13039/501100002301Estonian Research Council, Estonian Ministry of Education and Research, University of Tartu.

**Research in context**

**Evidence before this study**

The increased prevalence of metabolic syndrome among individuals with schizophrenia has undergone extensive scrutiny. To evaluate prior evidence of the underlying cause, we searched PubMed (MEDLINE) on January 17, 2024, using the search terms “schizophrenia” AND (“metabolic” OR “metabolic disorder” OR “body mass index”) OR (“antipsychotic” OR “antipsychotic treatment”) OR (“real-world data” OR “biobank”) OR (“genetic” OR “polygenic risk score”) for papers published in English until January 17, 2024. We identified several clinical and population-based studies and review articles that provide evidence of antipsychotics and genetics to metabolic alterations independently or examined the shared genetic architecture of these disorders using published summary-level data. While a few recent studies explored the simultaneous effect of treatment and genetics on the development of metabolic syndrome among schizophrenia cases, these were hindered by low sample size, lack of control group, and a short follow-up period (≤12 months). We found no longitudinal real-world evidence assessing their concurrent effect over extended follow-up.

**Added value of this study**

To our knowledge, this study is the first to comprehensively characterize antipsychotic use, genetic predisposition, and the prevalence of metabolic syndrome among individuals with schizophrenia over a ten-year follow-up period in a large-scale real-world healthcare setting. By leveraging longitudinal health records from the Estonian Biobank, comprising 200,000 participants, we unveil the significant impact of antipsychotic treatment and genetic predisposition on the development of metabolic syndrome and the trajectories of body mass index in individuals with schizophrenia. While higher adherence within the first year of treatment was linked with a reduced long-term incidence of metabolic disorders, the intensity of treatment contributed to the upward trend in body mass index among schizophrenia patients over time. This study underscores the value of longitudinal biobanks with extensive health records and genotype data as valuable resources for providing observational real-world evidence of the comorbidity of schizophrenia and metabolic syndrome.

**Implications of all the available evidence**

Suboptimal management of metabolic syndrome in schizophrenia patients contributes to increased mortality rates in this population, emphasizing the need for improved prediction and prevention strategies. Our study extends upon clinical and population-based research and highlights the role of genetics in the development of metabolic syndrome among schizophrenia patients, despite the inverse genetic correlation between schizophrenia and body composition traits. Our findings have implications for clinical guidelines (integration of genetic data for personalized treatment, early intervention, and close monitoring of metabolic outcomes), improved health initiatives (raising awareness of risks tied to antipsychotics and genetics, promoting preventive measures), and future research directions (integration of pharmacogenomics, longitudinal assessment of genetic and antipsychotic effects on clinical biomarkers in diverse populations).

## Introduction

Schizophrenia (SCZ) is a neurodevelopmental disorder affected by genetic and environmental risk factors with symptoms manifesting in early adulthood.^1^ SCZ patients have a 15-year shorter lifespan^2^ and exhibit 30% higher prevalence of metabolic syndrome (MetS) compared to the general population.^3^ As suboptimal management of the clustered symptoms of MetS (dyslipidaemia, abdominal obesity, hypertension, and hyperglycaemia) has a substantial impact on mortality among SCZ patients,^4^ improved understanding of the comorbid condition is warranted.

Increased prevalence of MetS among SCZ patients is attributed to the adverse effects of antipsychotic medications^5^ and lifestyle factors.^6^ Antipsychotics are linked with weight gain, abdominal obesity, and alterations in lipid and glucose metabolism with the most efficacious antipsychotics associated with the greatest metabolic disturbances.^5^ Research additionally shows a higher prevalence of MetS in first-episode antipsychotic-naïve psychosis patients^7^ and in unaffected relatives,^8^ suggesting a genetic component in MetS development in SCZ. Although genetic correlation analyses indicate an inverse relationship for SCZ with body mass index (BMI), obesity, and type II diabetes (T2D)^9^, ^10^, ^11^ and negligible associations with other cardiovascular traits,^11^ polygenic risk score (PRS) analyses reveal conflicting results between the genetics of SCZ and cardiometabolic traits with respective outcomes.^12^ Moreover, mendelian randomization studies implicate inflammation as a mediator of the associations.^13^ While the partially opposing findings can be driven by methodological differences, these collectively underscore intricate molecular mechanisms underlying the comorbidity of SCZ and MetS.

To assess the contribution of genetic predisposition and treatment effects to MetS development in SCZ, longitudinally followed patients with matched controls are needed. Despite a wealth of clinical and population-based studies,^14^, ^15^, ^16^, ^17^ these have been limited by low sample size, short follow-up, assessment of specific antipsychotics, or have not accounted for treatment and genetic effects simultaneously. While randomized control trials are the gold standard for studying causal relationships, these are hindered by strict eligibility criteria, low sample size, short-term follow-up, and limited external validity. Real-world (RW) data captured from large-scale biobanks linked with longitudinal health records could offer a feasible alternative for capturing observational long-term evidence.^18^

**Fig. 1:**
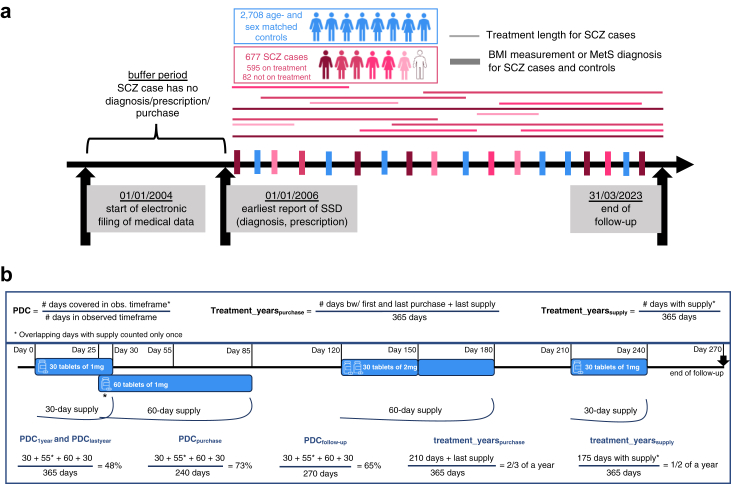
Overview of the study design (a) and the derivation of treatment variables from digital drug dispensing data (b). The colour darkness for SCZ cases in panel (a) indicates the length of treatment. Panel (b) outlines the formulas for deriving adherence and treatment length variables, accompanied by a mock example. SSD, Schizophrenia Spectrum Disorder.

To extend upon clinical and population-based research, we set out to assess the comorbidity of MetS and SCZ in a RW healthcare setting using extensive health records in the Estonian Biobank (EstBB).^19^ Specifically, to investigate the impact of genetic predisposition and antipsychotic treatment to MetS development in SCZ, we sought to characterize treatment patterns, genetic predisposition, MetS burden, and BMI trajectories over time comparing SCZ cases to age- and sex-matched controls (Fig. 1A).

## Methods

### Study cohort

EstBB is a longitudinal biobank in Northern Europe that holds genotype and phenotype data for 20% of the Estonian adult population.^19^ Details about defining the study cohort and variables of interest are outlined in the Supplementary Methods. In brief, SCZ cases had their first report of SCZ onset, i.e., either first antipsychotic prescription (ATC N05A∗, except lithium) or first Schizophrenia Spectrum Disorder (SSD) diagnosis (ICD-10 code F20-F29) at age ≥15 and ≤ 40 and after year 2006 to align with the start of the electronic filing of medical bills in Estonia in 2004 (Fig. 1A). For each SCZ case, four controls without any behavioural or mental disorders (ICD-10 F∗ codes) nor antipsychotic prescriptions were selected based on birth year and sex, using *R/MatchIt*^20^ with *method* = *nearest* parameter (Supplementary Figure S1). Digital drug dispensing data were used for deriving i) four metrics of adherence as the proportion of days covered (PDC) and considering different time windows (PDC_1year_, PDC_lastyear_, PDC_purchase_, PDC_follow-up_), ii) two variables for treatment length based on either days from first to last purchase (treatment_years_purchase_, unit years) or considering only the days with antipsychotic supply (treatment_years_supply_, unit years), and iii) the median chlorpromazine-equivalent antipsychotic dose (Fig. 1B, Supplementary Figure S2). The following antipsychotics were purchased: quetiapine, olanzapine, aripiprazole, clozapine, risperidone, haloperidol, amisulpride, sertindole, flupentixol, perphenazine, ziprasidone, levomepromazine, zuclopenthixol, chlorprothixene, sulpiride, cariprazine, melperone, chlorpromazine, fluphenazine, paliperidone. Data freeze 2023v1 with follow-up data until 31/03/2023 was used for analyses.

### Polygenic risk scores

All EstBB participants were genotyped with quality control conducted according to best practices and data imputed using the population-specific reference panel. PRS-cs^21^ was used for calculating PRSs for SCZ and 14 MetS traits (coronary heart disease (CHD), total (TC), LDL, HDL and nonHDL cholesterol, triglycerides (TG), C-reactive protein (CRP), glycated haemoglobin (HbA1c), fasting (FG) and random glucose (RG), T2D, BMI, systolic (SBP) and diastolic blood pressure (DBP)) using summary statistics from published genome-wide association studies (GWAS). Only independent variants at genome-wide significant threshold were used for PRSs of clozapine, norclozapine and their ratio (Supplementary Methods).

### Statistical analyses

For association testing between PRSs with disease status and with calculated median chlorpromazine-equivalent dose, multivariate regression analyses were performed in a forward stepwise manner. Namely, each PRS of interest was first modelled independently as an independent variable with baseline characteristics (sex, birth year, and 10 genotype PCs) as covariates (main model). Next, the PRSs were incrementally added to the main model, starting with the one with the lowest P-value and scanning through all other PRSs. Linear and logistic regression were used where appropriate. Significant associations were identified based on Bonferroni correction (0.05/15 = 0.0033).

Multivariate Cox proportional hazards modelling was applied to assess MetS incidence with PRSs modelled in a forward stepwise manner as described in the previous section. Bonferroni correction (0.05/6 = 0.0083) was applied to account for six endpoint-specific analyses.

To test the difference of BMI trajectories between cases and controls, linear mixed modelling was employed. Population parameters (sex, BMI PRS, 10 genotype PCs) were modelled as fixed effects and subject-specific effects (subject-specific random intercept and subject-specific age effect) as random effects. Firstly, we considered two models, a baseline model and a model that additionally included an interaction term between disease status and age at BMI measurement. To account for treatment effect, the model with better fit identified in the first approach was compared with a model where the quartic polynomial of treatment years was additionally included. Model fit was assessed with ANOVA. Eighty-three percent confidence intervals were used in main figures to denote statistically significant differences (alpha = 0.05) between two means with non-overlapping confidence intervals as recommended by Goldstein et al.^22^

Additional details are provided in the Supplement.

### Role of the funding source

The funders of the study had no role in study design, data collection, data analysis, data interpretation, or writing of the report.

## Results

### Cohort characteristics

To study treatment patterns and MetS burden, we applied numerous filtering criteria to account for the nature of RW data (Supplementary Methods, Supplementary Figure S1). The sample set (n = 3385) consisted of 677 SCZ cases and 2708 birth year- and sex-matched controls (54% female; Table 1, Supplementary Figure S3A). Information about smoking and BMI were available for >92% of the cohort (Supplementary Table S1). While smoking was more common among SCZ cases (P = 3.30 × 10^−11^, odds ratio (OR) 1.82; Supplementary Figure S3B), it was captured on average 3.7 years after SCZ onset (Supplementary Figure S3C). The majority had at least three BMI measurements (Supplementary Figure S3D and E) with a median interval of 332 days between measurements. Genotype data for analyses including PRSs was restricted to unrelated samples not included in previous GWAS.

**Table 1:** Overview of the study cohort.

Characteristic	SCZ cases	Controls
N of subjects	677	2708
Female	364 (53.8%)	1456 (53.8%)
Death over follow-up	13 (1.92%)	5 (0.18%)
Smoking ever	377 (55.7%)	1157 (42.7%)
Smoking never	248 (36.6%)	1385 (51.1%)
Smoking NA	52 (7.7%)	166 (6.1%)
BMI	mean 25.1; median 24.0 (SD 5.06)	mean 24.7; median 24.0 (SD 4.40)
N of BMI datapoints	mean 3.26; median 3 (SD 1.86)	mean 3.69; median 4 (SD 1.72)
On treatment	595 (87.9%)	
Age at disease onset	mean 27.0; median 26.0 (SD 6.89)	
Age at first AP purchase	mean 27.9; median 27.0 (SD 7.13)	
Follow-up from SCZ onset (years)	mean 10.2; median 10.5 (SD 4.58)	
Follow-up from first AP purchase (years)	mean 9.48; median 9.80 (SD 4.65)	
Count of AP purchases	mean 34.7; median 20 (SD 38.5)	
Count of different AP medications	mean 2.91; median 3 (SD 1.80)	
Calculated chlorpromazine-equivalent dose	mean 223.8 mg; median 186.0 mg (SD 174.8)	
PDC_1year_	mean 55.5%; median 54.8% (SD 32.3)	
PDC_lastyear_	mean 61.1%; median 71.5% (SD 35.6)	
PDC_purchase_	mean 63.6%; median 70.8% (SD 29.8)	
PDC_follow-up_	mean 43.7%; median 38.7% (SD 32.8)	
Treatment years_supply_ (in years)	mean 4.04; median 2.47 (SD 4.02)	
Treatment years_purchase_ (in years)	mean 6.87; median 6.24 (SD 5.15)	

Categorical variables are presented as frequencies (percentages), continuous variables with mean and median (standard deviation, SD). For BMI, mean and median were calculated across median BMI measurement values per individual. AP, antipsychotic.

### Treatment patterns of SCZ cases

Eighty-eight percent (n = 595) of SCZ cases purchased antipsychotics at least once (Supplementary Figure S4A and B) with the first drug dispensed shortly after the first prescription (Supplementary Figure S5A–D). On average, three different antipsychotic medications were purchased during follow-up (Table 1, Supplementary Figure S5E). No pattern of preference nor deviation by age group was observed (Supplementary Figure S5F and G), indicating lack of bias by purchase count and age at disease onset. The mean follow-up time for SCZ cases from disease onset (first SSD diagnosis or antipsychotic prescription) and from first antipsychotic purchase was 10.2 years (SD = 4.58) and 9.48 years (SD = 4.65), respectively (Supplementary Figure S6A and B).

Acknowledging that digital drug dispensing data do not reflect the ground truth of treatment, various approaches were applied to characterize antipsychotic treatment patterns for SCZ cases (Fig. 1B, Supplementary Figure S2). For medication adherence, we considered: i) adherence during the first treatment year (PDC_1year_), ii) during the last treatment year (PDC_lastyear_), iii) over antipsychotic purchases (PDC_purchase_), and iv) over follow-up (PDC_follow-up_). PDC_purchase_ captured the highest adherence rate (mean 63.6%) and PDC_follow-up_ the lowest (mean 43.7%; Table 1, Supplementary Figure S7A–D). The derived adherence metrics exhibited varying levels of correlation depending on purchase count and treatment length (Supplementary Figure S8A–C). Among SCZ cases who dispensed antipsychotics >1 year (n = 480), PDC_lastyear_ showed stronger correlations with PDC_purchase_ and PDC_follow-up_ (ρ = 0.75) than with PDC_1year_ (ρ = 0.40), while PDC_purchase_ exhibited similar correlations across all adherence variables (ρ = 0.69–0.89; Supplementary Figure S8C).

Despite the strong correlation between treatment length variables (ρ = 0.83), i.e., from first to last purchase (treatment_years_purchase_) vs based on days with antipsychotic supply (treatment_years_supply_), the large difference in medians (6.24 vs 2.47 years) indicated considerable treatment gaps for SCZ cases (Table 1, Supplementary Figure S9A–C). Forty-one percent (243 of 595) of SCZ cases stopped treatment >1 year before the end of follow-up/death (Supplementary Figure S9D and E) with discontinuation occurring at a median of 2.10 years after first purchase (Supplementary Figure S9F). Among those who dispensed antipsychotics until the end of follow-up/death, 57% (200 of 352) had >8 years between first and last purchase (Supplementary Figure S9G). While 44 (7%) of SCZ cases never purchased metabolically more active antipsychotics, 75% of SCZ cases purchased these for at least half of their treatment duration (Supplementary Figure S9H and I).

The calculated median chlorpromazine-equivalent antipsychotic dose for SCZ cases was 223.8 mg on average (SD = 174.8; Supplementary Figure S10A). It was positively associated with PDC_purchase_ (β = 0.48, SE = 0.06, P = 1.27 × 10^−13^) and with treatment_years_supply_ (β = 0.03, SE = 0.005, P = 3.96 × 10^−9^) in a model that additionally accounted for birth year and sex.

### Polygenic predisposition of MetS traits

**Fig. 2:**
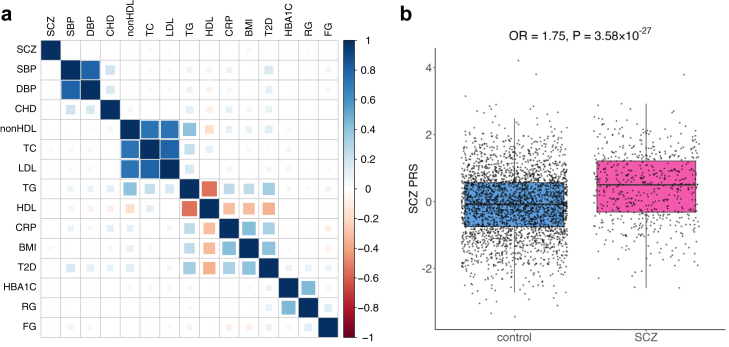
PRSs of MetS traits and SCZ. (a) Correlation matrix of the PRS for 14 MetS traits and SCZ. (b) Boxplot of SCZ PRS distribution for SCZ cases and controls.

To assess the genetic liability to MetS, PRSs for 15 traits (CHD, TC, LDL, HDL, nonHDL, TG, CRP, Hb1Ac, fasting glucose (FG), random glucose (RG), T2D, BMI, SBP, DBP, and SCZ) were considered. As expected, PRSs for blood pressure (SBP, DBP), lipids (TC, LDL, nonHDL vs HDL, TG) and glycaemic traits (Hb1Ac, RG vs FG) clustered in distinctive groups (Fig. 2A). Notably, the PRSs for TG, T2D, CRP, BMI, and HDL grouped together with the former four PRSs being positively correlated and HDL displaying a negative correlation. Of note, the CRP PRS exhibited a positive correlation of 0.40 with BMI PRS and 0.32 with T2D PRS and HbA1c showed a stronger correlation with random glucose (ρ = 0.43) than with fasting glucose (ρ = 0.05). The patterns were consistent when considering all unrelated EstBB participants and the genetic correlation matrix based on GWAS (Supplementary Figure S11A and B) and thus align with the shared genetic basis of biologically similar MetS traits.^23^

In a multivariate logistic regression analysis, only SCZ PRS was significantly associated with disease status after multiple testing correction (OR = 1.75, 95% confidence interval (CI) 1.58–1.94, P = 3.58 × 10^−27^; Fig. 2B). PRSs for BMI, CRP, HDL, nonHDL, T2D, and DBP were nominally associated when modelled independently (Supplementary Table S3, Supplementary Figure S11C and D). None of the PRSs for SCZ, MetS or clozapine and its metabolite levels associated nominally with median chlorpromazine-equivalent dose.

### Incidence of MetS

Compared to controls, SCZ cases displayed significantly earlier disease onset for T2D (HR = 4.36, 95% CI 1.96–9.68), essential hypertension (HR = 1.91, 95% CI 1.41–2.60), CHD (HR = 3.31, 95% CI 1.62–6.78), CVD (HR = 3.45, 95% CI 1.80–6.62), and any metabolic disorder (HR = 1.94, 95% CI 1.54–2.45) and nominally for hypercholesterolemia (HR = 1.42, 95% CI 1.05–1.93; Table 2). Notably, PRS for DBP and PRSs for DBP, and nonHDL exceeded multiple testing threshold for essential hypertension and for any metabolic disorder, respectively. Endpoint-specific PRSs showed nominal significance for T2D, hypercholesterolemia, CHD, and CVD when considered independently (Table 2, Supplementary Figure S12).

**Table 2:** Overview of survival analyses.

Endpoint	N^a^	N of incident endpoints	HR (95% CI) for SCZ	PRSs surviving multiple testing	PRSs only nominally significant^b^
T2D	528 SCZ 2511 controls	14 SCZ 12 controls	4.19 (1.88–9.32)	None	T2D, HbA1c, HDL, nonHDL, TG
Hypercholesterolemia	525 SCZ 2479 controls	58 SCZ 188 controls	1.43 (1.06–1.94)	None	CHD, LDL, TC, nonHDL, TG
Essential hypertension	494 SCZ 2396 controls	60 SCZ 151 controls	1.92 (1.41–2.61)	DBP	SBP, CHD, BMI, T2D, HDL
CHD	531 SCZ 2511 controls	14 SCZ 19 controls	3.35 (1.64–6.84)	None	nonHDL
CVD	530 SCZ 2510 controls	17 SCZ 23 controls	3.48 (1.81–6.67)	None	nonHDL
Any metabolic disorder	485 SCZ 2343 controls	103 SCZ 280 controls	1.95 (1.54–2.46)	DBP, nonHDL	SBP, CHD, T2D, TG

a N of SCZ cases and controls considered in endpoint-specific analyses.

b PRSs considered independently in the model.

Despite the moderate correlation of PDC_1year_ with other PDC metrics (Supplementary Figure S8), better adherence during the first medication year could contribute to long-term improvement in (co-morbid) disease progression.^24^^,^^25^ To investigate this, we assessed the association of PDC_1year_ with MetS incidence among SCZ cases. PDC_1year_ was nominally associated with lower incidence of essential hypertension (HR = 0.69, 95% CI 0.50–0.96) and any metabolic disorder (HR = 0.77, 95% CI 0.60–0.99) with none of the PRSs surviving multiple testing (Supplementary Table S4).

### BMI trajectories in SCZ cases and controls

**Fig. 3:**
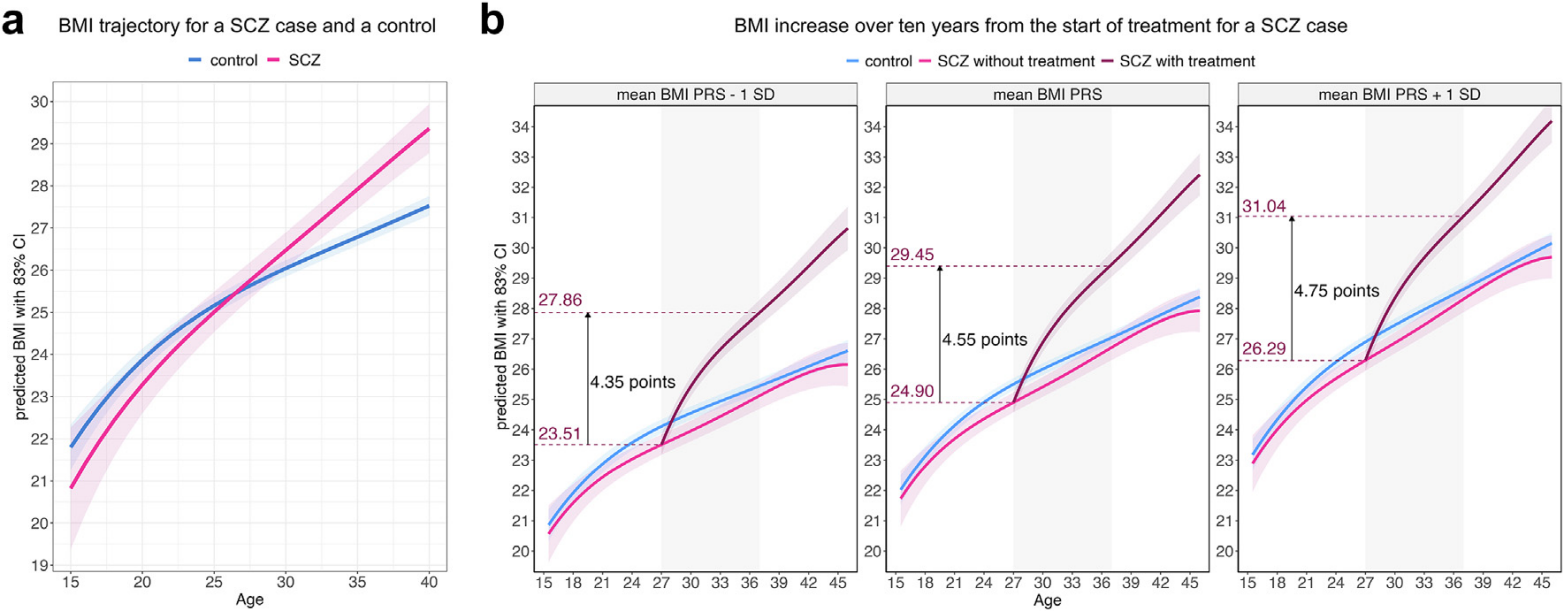
Predicted BMI trajectories over time with 83% confidence intervals. (a) The predicted BMI trajectory over time for a SCZ case is significantly different compared to an unaffected individual. In the prediction model, a male SCZ case and a male control with smoking history and with a BMI PRS equalling to the population average are considered. (b) Predicted BMI trajectories over time for a male SCZ case on treatment (dark pink), for a male SCZ case not on treatment (pink) and for an unaffected male individual (blue) by BMI PRS (BMI PRS 1 SD lower than the population average, BMI PRS equal to the population average, BMI PRS 1 SD higher than the population average). BMI increase over a decade is outlined for a male SCZ case taking antipsychotics 180 days every year starting from age 27 with the bottom dotted line indicating the BMI value at the initiation of antipsychotic treatment and the top dotted line denoting the BMI value after ten years of treatment. CI, confidence interval.

To capture BMI trajectories over time, we accounted for multiple BMI measurements per individual and additionally considered BMI PRS given its nominally significant negative association with SCZ (Supplementary Figure S11C, Supplementary Table S3). The model with the interaction term for disease status and age at BMI measurement provided better fit compared to baseline model (ANOVA P = 2.76 × 10^−8^), denoting a significantly different BMI trajectory over time for SCZ cases compared to controls (Fig. 3A, Supplementary Figure S13A, Supplementary Table S5).

To delineate whether this difference was driven by antipsychotic treatment, we considered treatment years based on supply up to each BMI measurement. The model including treatment length showed better fit (ANOVA P = 1.26 × 10^−25^), whereby SCZ cases on treatment displayed a significantly different BMI trajectory over time compared to controls and to SCZ cases without treatment. The latter group showed a lower BMI trajectory compared to controls (Fig. 3B, Supplementary Figure S13B, Supplementary Table S6). Specifically, over a decade, an unaffected individual and an untreated SCZ case would gain on average 1.5 and 1.8 BMI points, respectively. In contrast, BMI increase for a SCZ case taking antipsychotics 180 days every year would be 4.6 BMI points (Supplementary Figure S14). Moreover, at treatment initiation, a SCZ case with high BMI PRS starts at a BMI level 0.61 points lower than a control with an equivalent BMI PRS and reaches the same BMI level in one year of treatment. Conversely, a SCZ case with low BMI PRS starts at a BMI level that is 3.4 points lower than that of a control with high BMI PRS and reaches the same BMI in 15 years of treatment (Supplementary Figure S15).

Lastly, to investigate the impact of treatment on BMI increase, we focused on SCZ cases and considered the median chlorpromazine-equivalent dose and adherence assessed two years prior to each BMI measurement and treatment years based on supply information up to each BMI timepoint. The median dose and adherence captured two years prior to BMI measurements showed strong correlations with median dose and PDC_purchase_ assessed over the entire antipsychotic supply period (ρ = 0.76 and ρ = 0.63, respectively, Supplementary Figure S16), indicating absence of systematic over- or underestimation in treatment variables using the two-year timeframe. The upward BMI trajectory observed among SCZ cases under treatment was significantly affected by median dose (β = 1.18, SE = 0.38), adherence (β = 1.40, SE = 0.53), treatment duration (β = 0.27, SE = 0.08) and BMI PRS (β = 1.56, SE = 0.29; Supplementary Table S7).

## Discussion

The increased prevalence of MetS among individuals affected by SCZ has been linked with antipsychotics, genetic predisposition, and lifestyle factors. Despite extensive research, longitudinal large-scale studies accounting for all these factors remain scarce. To address this gap, we used longitudinal RW health data from EstBB to investigate the impact of genetics and treatment on MetS development in SCZ. This approach enabled us to select age- and sex-matched controls from the general population, characterize treatment patterns over a median follow-up of ten years and assess the burden of MetS in relation to its genetic liability. Our findings revealed a significantly higher prevalence of MetS and an upward BMI trajectory over time among SCZ cases compared to unaffected individuals, with a substantial contribution from polygenic predisposition. While the markedly different BMI trajectory was driven by the duration, dosage, and adherence to treatment, higher adherence during the first treatment year was associated with reduced long-term incidence of MetS.

This study is among the first to comprehensively scrutinize MetS development in SCZ within a large-scale longitudinal RW setting. Specifically, we observed that SCZ cases exhibited significantly higher genetic predisposition to SCZ, as expected, but showed lower polygenic burden for BMI and CRP compared to controls. These findings align with prior research underscoring an inverse relationship between the genetic liabilities for body composition traits, T2D, and inflammation with SCZ.^9^^,^^11^, ^12^, ^13^ The positive correlation between BMI PRS and CRP PRS supports the proposition that MetS deviations could partly be mediated through inflammatory processes.^13^

While we noted that SCZ cases displayed worse prognoses for MetS-related endpoints compared to controls with a notable impact from genetic predisposition to MetS, higher adherence during the first treatment year was linked to improved long-term MetS prognosis. Previous studies relying on RW health data show similar results, indicating high rates of incident CVD events and MetS in SCZ,^26^^,^^27^ that SCZ patients with T2D display higher genetic predisposition to both disorders compared to controls^28^ and positive familial aggregation,^29^ and that better adherence to antipsychotic treatment regime associates with lower all-cause and CVD mortality rates and with lower discontinuation of MetS medications.^24^^,^^25^ Better adherence during the first treatment year may be indicative of increased readiness for improved management of SCZ symptoms, lifestyle choices, and antipsychotic treatment^24^^,^^25^ and underscores that greater engagement with the medical system provides the means to improve primary and secondary prevention of MetS in this population. Given the multifaceted nature of adherence, influenced by subjective perception and disease-specific factors, further research efforts are needed to enhance patients' readiness to adhere to treatment and empower them to manage their health and well-being effectively.

We observed that the difference in BMI trajectory over time (lower at younger age, but steeper slope at older age for SCZ cases) was significantly augmented by both antipsychotic treatment, and genetic predisposition to BMI. Furthermore, the upward trajectory among SCZ cases was driven by better adherence, higher antipsychotic dose, and longer treatment duration. Notably, the antipsychotics associated with the greatest metabolic disturbances (olanzapine, clozapine, quetiapine^5^) were the most purchased drugs in the current study (Supplementary Table S2). While these results align with existing research linking weight gain to the duration and dosage of antipsychotic use,^30^^,^^31^ and similar point estimates over a decade,^16^ we further ascertain that the genetic liability to increased BMI predisposes to an upward trajectory beyond antipsychotic treatment and corroborate the value of including BMI PRS for trait prediction at treatment initiation.^15^ Furthermore, while still in its infancy, ongoing analysis on microbiome in SCZ^32^ can provide further clarification of MetS development among these patients. These endeavours open new perspectives for personalized antipsychotic pharmacotherapy.

While large-scale biobanks are valuable resources for investigating clinical research questions, especially given their longitudinal follow-up, lack of selection bias, and availability of standardized healthcare data, rigorous approaches are required to recognize and address potential biases inherent in such resources. To this end, the strengths of this study must be assessed alongside the limitations. Firstly, we used a two-year buffer period from the start of the electronic filing of medical data in Estonia until the first report of SCZ onset to ensure that we captured treatment trajectories from the beginning of treatment intervention for SCZ cases. However, given the partial mismatch between the dates of SCZ diagnosis and first antipsychotic prescription, unavailability of medication information provided during hospitalizations, limited data on outpatient procedures, and the possibility that a treatment gap coincided with buffer period, we cannot rule out that some patients could have received treatment before the start of follow-up.

Secondly, to characterize treatment patterns, we acknowledged the limitations of drug dispensing data, which outline purchase patterns rather than true treatment regimes, and lack the information on drug intake and exact timing of treatment discontinuation. We examined four different adherence variables that captured either the first or last treatment year, the time between all purchases or over follow-up and observed variation in correlation patterns. Considering variation in the timing of treatment initiation among SCZ patients and the lifelong nature of antipsychotic treatment, the PDC_lastyear_ variable provided a cross-sectional estimate that strongly correlated with adherence metrics assessed over the duration of treatment or follow-up. The moderate correlation between PDC_1year_ and PDC_lastyear_ reflects that adherence assessed within the first year is not always reflective of treatment characteristics of SCZ cases in general. A one-year cross-sectional assessment can provide a better overview of treatment patterns if longer follow-up data per individual are available, emphasizing the need for longitudinal studies extending beyond the typical twelve-month duration applied in clinical studies. Additionally, given that pharmacy claims do not indicate whether the medication dispensed was taken as prescribed, the derived PDC variables could have potentially overestimated true adherence. To account for the reluctance of SCZ cases to adhere to treatment regimes, we considered two different treatment length variables. Treatment_years_purchases_ covered the entire duration between the first and last purchase, while treatment_years_supply_ considered only days with actual supply. The difference in their median length highlighted significant treatment gaps for SCZ cases, attributable to hospitalizations, extended periods abroad, or true breaks in treatment. To account for these gaps, we considered purchases in batches when deriving the median antipsychotic dose.

Thirdly, while we considered smoking and BMI as proxies for lifestyle factors in survival models, the report date did not explicitly match disease onset for SCZ cases and matched controls. To minimize any bias, we considered BMI ranges rather than real values and ever vs never smoking history. However, this could have introduced some residual confounding. Other environmental risk factors such as diet, physical activity levels, substance use, early life stress events, and birth weight^33^ are also crucial to be assessed in a longitudinal context to fully understand their impact on antipsychotic-induced weight gain and MetS among individuals with SCZ. Fourth, pooled data on all antipsychotics prevented from accounting for pharmacogenomic profiles and potentially contributed to the lack of association between antipsychotic dose and the genetic liability of assessed traits. Given previous research associating SCZ PRS with clozapine use,^34^ future studies with larger sample sets incorporating pharmacogenomics may clarify this. Such cohorts would also allow deeper study of the effects and interactions of PRS for specific drug concentration levels and genetic variants in known respective drug metabolizing enzymes. Furthermore, the use of multiple medications throughout patients' treatment course and reduced power when restricting to single medication users (Supplementary Figure S5E) precluded analysis by antipsychotic stratification. Larger samples may enable modelling antipsychotics individually. Fifth, due to modest availability of additional MetS-associated clinical variables, particularly those significantly affected by MetS medications, we opted against depicting partial trajectories for glucose, glycated haemoglobin, and lipid variables. Instead, we chose BMI as a proxy for MetS, given its robust and wide availability in RW data, strong association with MetS deviations,^35^ and its lesser susceptibility to unknown technical and biological variables. Lastly, given the voluntary enrolment to EstBB, the studied cohort of SCZ cases and controls might not be representative of the whole population.

In conclusion, we have elucidated the significance of leveraging RW health data to characterize treatment patterns, MetS burden, and its genetic predisposition among SCZ patients. We underscore the importance of incorporating genetic information, tracking patients over long follow-up, and using multiple angles to account for inherent characteristics of claims data. Our findings provide further evidence to enhance clinical risk prediction and prevention strategies for MetS among SCZ patients.

## Contributors

Conceptualisation: MA, LM. Data curation: MA, SK, LBL, EBRT, HIRT. Formal analysis: MA, SK, LBL. Funding acquisition: LM. Investigation: MA, SK, LBL, LH. Methodology: MA, SK, LBL, KF, MM. Supervision: MA, LM. Visualisation: MA, SK, LBL. Writing: original draft: MA, SK, LH, LM. Writing—review & editing: MA, SK, LH, LBL, KF, MM, LM.

## Data sharing statement

GWAS summary statistics are publicly available as provided in respective publications. Individual-level data at EstBB can only be accessed through EstBB. Further info about access is provided at https://genomics.ut.ee/en/content/estonian-biobank.

## Declaration of interests

All authors declare no competing interests.
