# Supplementary material for: Genetic predisposition and antipsychotic treatment effect on metabolic syndrome in schizophrenia: a ten-year follow-up study using the Estonian Biobank
Source: Lancet Reg Health Eur. 2024 Apr 26;41:100914. doi: 10.1016/j.lanepe.2024.100914 (PMC11066665; doi:10.1016/j.lanepe.2024.100914)
Supplement: Supplementary Figures and Tables [file mmc1.pdf]

Supplementary Information

Genetic predisposition and antipsychotic treatment effect on metabolic syndrome in schizophrenia: a ten-year follow-up study using the Estonian Biobank

Maris Alver PhD[1], Silva Kasela PhD[1], Liina Haring MD[2,3], Laura Birgit Luitva MSc[1], Estonian Biobank Research Team[1], Health Informatics Research Team[4], Krista Fischer PhD[1,5], Märt Möls PhD[1,5], Lili Milani PhD[1]

1) Estonian Genome Centre, Institute of Genomics, University of Tartu, 23b Riia Street, Tartu, 51010, Estonia
2) Department of Psychiatry, Institute of Clinical Medicine, University of Tartu, 31 Raja Street, Tartu, 50417, Estonia
3) Psychiatry Clinic of Tartu University Hospital, 31 Raja Street, Tartu, 50417, Estonia
4) Institute of Computer Science, University of Tartu, Narva mnt 18, Tartu, 51009, Estonia
5) Institute of Mathematics and Statistics, University of Tartu, Narva 18, Tartu, 51009, Estonia

This PDF file includes:
Supplementary Methods
References for Supplementary Methods
Supplementary Figures 1 to 18
Supplementary Tables 1 to 7

TABLE OF CONTENTS

SUPPLEMENTARY METHODS 4

Study cohort 4
Estonian Biobank (EstBB) 4
Selection of SCZ cases 4
Selection of controls 4
Smoking and BMI data 4

Derivation of treatment-associated variables 5
Median dose 5
Adherence 5
Treatment years 6

Genotype data and polygenic risk scores 6

Association testing and statistical analyses 7
Association testing between median chlorpromazine-equivalent dose and treatment variables 7
Association testing between PRSs with disease status and the median chlorpromazine-equivalent dose 7
Survival analyses 7
BMI trajectory analyses 7

References for Supplementary Methods 8

SUPPLEMENTARY FIGURES 10

Supplementary Figure 1. Flowchart for selecting SCZ cases and birth year- and sex-matched controls in EstBB. 10

Supplementary Figure 2. Overview of the derivation of treatment variables from digital drug dispensing data 11

Supplementary Figure 3. Smoking and BMI data. 12

Supplementary Figure 4. Distributions of antipsychotic purchases. 13

Supplementary Figure 5. Overview of antipsychotic purchases. 15

Supplementary Figure 6. Follow-up of SCZ cases. 16

Supplementary Figure 7. Distributions of adherence variables. 17

Supplementary Figure 8. Spearman correlations among adherence variables. 18

Supplementary Figure 9. Treatment years variables. 20

Supplementary Figure 10. Median dose. 21

Supplementary Figure 11. PRSs of MetS traits and SCZ. 22

Supplementary Figure 12. Forest plots of endpoint-specific survival analyses. 23

Supplementary Figure 13. Distribution of raw BMI measurement values. 24

Supplementary Figure 14. Predicted BMI trajectories over time modelled for a SCZ case using different levels of antipsychotic supply per year in relation to a SCZ case not on treatment and a control individual. 25

Supplementary Figure 15. Predicted BMI trajectories over time for control with high BMI PRS and a SCZ case with different BMI PRS values. 26

Supplementary Figure 16. Comparison of median dose and adherence values derived using different time scales 27

Supplementary Figure 17. Report of the Schizophrenia Spectrum Disorder diagnoses in EstBB. 28

Supplementary Figure 18. Correlations between treatment variables and calculated median chlorpromazine-equivalent dose. 29

SUPPLEMENTARY TABLES 30

Supplementary Table 1. Overview of the availability of smoking, BMI, and genotype data for SCZ cases and controls. 30

Supplementary Table 2. Overview of the purchased antipsychotics by SCZ cases (n=595) in EstBB data. 31

Supplementary Table 3. Results of the association testing between individual PRSs and disease status. 32

Supplementary Table 4. Overview of the results of survival analyses with adherence captured within the first treatment year (PDC_{1year}) as a dependent variable. 33

Supplementary Table 5. Overview of the considered linear mixed models and the results for assessing BMI trajectories over time between SCZ cases and controls. 34

Supplementary Table 6. Overview of the considered linear mixed models and the results for assessing BMI trajectories over time between SCZ cases and controls while accounting for treatment length. 35

Supplementary Table 7. Overview of the linear mixed model and the results for assessing the BMI trajectory over time for SCZ cases while accounting for treatment-associated factors. 36

SUPPLEMENTARY METHODS

Study cohort

Estonian Biobank (EstBB)

Health records at EstBB were extracted from the data provided by regional hospitals, national registries, and the Estonian Health Insurance Fund, a national administrative database that pools detailed and individual-level billing data for all health care services as well as digital prescription information. Disease diagnoses are recorded based on the International Classification of Diseases, 10th revision (ICD-10 codes) and prescribed medication according to the Anatomical Therapeutic Chemical (ATC) classification system. The activities of EstBB are regulated by the Human Genes Research Act, which was adopted in 2000 specifically for the operations of EstBB. All EstBB participants have signed a broad informed consent. The study was approved by the Estonian Committee on Bioethics and Human Research at the Estonian Ministry of Social Affairs (24 March 2020, nr 1.1-12/624) and carried out using data according to release S47 from EstBB. Data freeze 2023v1 with follow-up data until 31/03/2023 at EstBB was used for analyses[1,2].

Selection of SCZ cases

SCZ cases were defined as individuals with Schizophrenia Spectrum Disorder (SSD), according to ICD-10[3] codes F20-F29. These encompass several conditions: schizophrenia, the central diagnosis of the group, schizotypal disorders, persistent delusional disorders, and a larger group of acute and transient psychotic disorders. While there were 2,473 individuals with the SSD diagnosis in data freeze 2023v1, we excluded individuals i) with no available medication prescription data, ii) with a bipolar disorder diagnosis confirmed by a neurology or a psychiatry specialist, iii) whose earliest diagnosis age was <15 and >40, and iv) who had their first report of SCZ onset (i.e., either received their first antipsychotic prescription or their first SSD diagnosis) before year 2006. The latter filtering criterium was applied due to two observational characteristics of biobank data. First, some SCZ cases received their disease diagnosis after antipsychotic prescription (Supplementary Figure 17A). Since the primary objective was to track patients from treatment initiation, treatment trajectories had to be captured from treatment onset and not from disease diagnosis. Hence, the earliest follow-up start date was set at either the date of the first diagnosis or the date of the first prescription. Second, considering that the electronic filing of medical bills within the Health Insurance Fund started in 2004, a buffer period of two years was introduced. This was applied to ensure that individuals who received their first SSD diagnosis or began their antipsychotic treatment prior to the implementation of the electronic filing system were excluded (Supplementary Figure 17B). All defined SCZ cases (n = 677) were of European ancestry. In analyses using PRSs, we excluded i) samples who were included in the GWASs used for calculating PRSs considered in the current study, and ii) one member per related pairs of SCZ cases (PLINK PI_HAT >0.2), totalling 577 individuals. The flowchart of selecting SCZ cases is provided in Supplementary Figure 1.

Selection of controls

To select age- and sex-matched controls, the following individuals were considered: i) individuals without any behavioural or mental disorders (ICD-10 F* codes), ii) who had not been prescribed antipsychotic medications (ATC N05A* codes), iii) who were not related to any individuals with SSD in EstBB or other potential controls (PLINK PI_HAT <0.2), iv) had not been included in sample sets of GWAS used for calculating PRSs considered in the current study, and v) were of European ancestry, totalling 43,969 individuals. Four birth year- and sex-matched controls were defined for each SCZ case using R/*MatchIt*[4] with the *method = nearest* parameter. This approach yielded four controls for each SCZ case of the same sex and born in the same year. The flowchart of selecting controls is provided in Supplementary Figure 1. Data about sex was retrieved from the information reported at participant enrolment to EstBB.

Smoking and BMI data

For SCZ cases and controls, smoking status (*ever/never*) was derived based on data reported at participant enrolment: *ever* – in case *former smoking* or *current smoking* was marked, *never* – in case *never smoking* was reported. BMI information was extracted from all available electronic health records (including data extraction from free-text sections in medical case summaries) linked with EstBB. Measurements <15 and >50 BMI points were excluded (79 of 13,122 datapoints). Consecutive BMI measurements were required to be >2 months apart (943 datapoints omitted). We additionally excluded: i) datapoints of individuals whose difference in consecutive weight measurements was >15kg within a period of less than 2 years and not supported by later

measurements, and ii) self-reported BMI measurements not supported by preceding and succeeding objective measurement data (17 datapoints omitted). Thus, after quality control, 2,163 and 9,920 BMI measurements were available for 664 SCZ cases and 2,691 controls, respectively. In survival analyses where BMI was considered as a covariate, the measurement closest to the entry date of the survival study was used. As the mean difference between the study entry date and the BMI measurement was 1,622 days (minimum 0, maximum 5,832 days, Supplementary Figure 3F), four BMI categories, i.e., (i) underweight (≤18.5), ii) normal weight (>18.5 and ≤25), iii) overweight (>25 and ≤30), and iv) obese (>30)), were used instead of real values.

Derivation of treatment-associated variables

Median dose

Throughout the follow-up period, 20 different types of antipsychotic medications with different routes of administration (oral, short-acting and long-acting injections) and with patterns of concurrent use were purchased (Supplementary Table 2). To this end, the Delphi method[5–7] was applied to standardize the doses of purchased antipsychotics to an equivalent dose of 100 milligrams (mg) per day of chlorpromazine. Specifically, the conversion factors outlined in Gardner et al.[7] were preferred and if missing (e.g., for short-acting injectables and for cariprazine and melperone), the dose-adjustment factors from Leucht et al.[8,9] were applied (R/*chlorpromazineR*)[10]. Long-acting injectables were first converted to the daily dose based on the minimum duration recommendation (i.e., 1 ampoule of 50 mg/ml of haloperidol recommended for a minimum of 14 days were converted to the daily dose equivalent of 187 mg of chlorpromazine for two weeks). To derive the median antipsychotic dose for SCZ cases, digital drug dispensing data for oral and long-acting antipsychotics (ATC N05A* codes except lithium) from the Estonian National Health Insurance Fund were used. The medicines in the digital drug dispensing data belong to the list of medicines reimbursed by the Estonian Health Insurance Fund and can be purchased at the pharmacy. The data consist of information regarding the date of drug prescription and purchase, the dose and content specified on each package, and the number of packages bought. We additionally considered outpatient procedures for long-acting antipsychotics provided by a psychiatric nurse, either for i) risperidone, olanzapine, aripiprazole, paliperidone, or for ii) perphenazine, fluphenazine based on billing data stored from 2015 onwards. These outlined long-acting antipsychotics are reimbursed centrally by the Estonian Health Insurance Fund and are administered to patients as outpatient treatment services in psychiatric clinics. Such long-acting injections were converted to an equivalent dose of 300 mg per day of chlorpromazine for 4 weeks and were considered as purchases. Specifically, for each purchase, the package content (i.e., dose in mg and the number of pills in a package) was multiplied by the number of packages bought. Purchases within two weeks were consolidated, such that the purchase date for all consecutive purchases were assigned to the date of the first 14-day purchase. Purchases separated by >365 days were considered as distinct purchase batches. Next, the calculated *mg × pills in a package × number of packages* variable for each purchase was divided by the number of days until the next purchase resulting in an estimate of a daily dose per purchase. Individual purchases or batch purchases consisting of a single purchase were excluded. Finally, the daily antipsychotic dose was derived by taking the median across the derived daily doses. In case different purchase batches were derived (>365 days between purchases), the median dose was calculated for each purchase batch, followed by a subsequent median dose calculation across all batches. Of note, the correlation with the widely used Defined Daily Dose method[11] was $\rho = 0.98$ (Supplementary Figure 10C).

Adherence

Four different adherence variables were derived using digital drug dispensing data for antipsychotics (ATC codes N05A* except lithium) and outpatient procedures for long-acting antipsychotics from the Estonian National Health Insurance Fund. Firstly, the proportion of days covered within the first treatment year (PDC_{1year}) was calculated to provide a uniform estimate of adherence during the initial year of treatment. The PDC_{1year} variable was derived for SCZ cases whose first antipsychotic prescription was dispensed at least one year before the end of follow-up (31/03/2023; n = 582; individuals who died within their first treatment year were excluded). It was calculated by counting the days with antipsychotic supply for all purchased antipsychotics within a year from the first purchase and divided by 365 days. Secondly, the proportion of days covered within the last available treatment year ($PDC_{lastyear}$) allowed to capture a cross-sectional estimate of adherence over a uniform period not affected by treatment initiation bias. The $PDC_{lastyear}$ variable was derived for SCZ cases who had purchase information available for more than one year (n = 480). It was calculated by determining the days' supply for all purchased antipsychotics within their last available treatment year (starting 365 days before the last antipsychotic purchase) divided by 365 days. The median duration of treatment until the beginning of the last treatment year was 6.96 years (interquartile range 7.94). Thirdly, the proportion of days covered over the study period ($PDC_{follow\text{-}up}$) was calculated over follow-up. The $PDC_{follow\text{-}up}$ variable was derived for all SCZ cases who had purchased antipsychotics at least once (n = 595) by calculating the days' supply for all purchased antipsychotics from the first purchase until the end of follow-up or death divided by the number of days between the first purchase and the end of follow-up or

death. Lastly, the proportion of days covered over the dispensing period ($PDC_{purchase}$) captured the time from first to the last purchase. The $PDC_{purchase}$ variable was derived for all SCZ cases who had purchased antipsychotics at least once (n = 595) by calculating days' supply for all purchased antipsychotics from the first purchase until the end of supply of the last purchase or death and divided by the number of days between the first purchase and the end of supply or death. If the end of supply of the last purchase extended beyond the follow-up end date, the end of supply was truncated to the end date of the follow-up period. For all adherence variables, the days' supply was calculated by multiplying the number of pills in a package by the number of packages bought per purchase, assuming one pill per day. Similarly to the median dose calculation, purchases within 14 days were consolidated, such that the purchase date of all consecutive purchases were assigned to the date of the first 14-day purchase. Purchased long-acting injectables were first converted to daily treatment based on the minimum duration recommendation. Long-acting injections provided by a psychiatric nurse were converted to daily treatment for 4 weeks.

Treatment years

Two different variables were derived to capture treatment years. Firstly, we counted the days from the first to the last purchase, added the supply of the last purchase and converted the summed days to a year ($treatment\_years_{purchase}$). Secondly, we added up the days' supply for each purchase and converted the number to years ($treatment\_years_{supply}$). These two variables offer different perspectives on treatment duration, the former focusing on the duration between the first and the last antipsychotic purchase, and the latter considering only the days with antipsychotic supply acquired during treatment. The proportion of metabolically more active antipsychotics taken over treatment years (i.e., $treatment\_years_{supply}$) was calculated by adding up the days' supply for clozapine, risperidone, quetiapine, and olanzapine and divided by $treatment\_years_{supply}$. Metabolically more active drugs were defined based on the British Association for Psychopharmacology (BAP) guidelines[12]. SCZ cases who received antipsychotics via outpatient procedures for long-acting antipsychotics (n=47) were excluded from this calculation.

Overview of the derivation of the treatment variables are depicted in Supplementary Figure 2 and the Spearman correlations of the calculated chlorpromazine-equivalent dose with adherence and treatment length variables in Supplementary Figure 18.

Genotype data and polygenic risk scores

All EstBB participants were genotyped using Illumina GSAv1.0, GSAv2.0, and GSAv2.0_EST arrays with quality control conducted according to best practices (exclusion of individuals with call rate <95%, with mismatch between genotype and phenotype sex and who deviated ±3SD from the samples' heterozygosity rate mean; exclusion of SNVs with call rate <95%, HWE p<1e-4, MAF <1%)[13]. Pre-phasing was carried out with Eagle v2.3[14] and imputation with Beagle[15] v.28Sep18.79367 using the population-specific imputation reference panel built from 2,297 whole genome sequencing samples[16]. One member per related individual pairs (PLINK PI_HAT >0.2) were excluded in association testing where PRSs were considered[13,17].

For PRS calculation, GWAS summary statistics based on GRCh37 for SCZ[18] and 14 MetS-associated traits (coronary heart disease (CHD)[19], total cholesterol (TC)[20], LDL-cholesterol (LDL)[20], HDL-cholesterol (HDL)[20], nonHDL-cholesterol (nonHDL)[20], triglycerides (TG)[20], C-reactive protein (CRP)[21], glycated haemoglobin (HbA1c)[22], fasting glucose (FG)[22], random glucose (RG)[23], type II diabetes (T2D)[24], body mass index (BMI)[25], systolic blood pressure (SBP)[26], and diastolic blood pressure (DBP)[26]) were considered with European-specific results preferred when available. SNVs with imputation score <0.8, MAF <0.01, HWE P<1e-4, and ambiguous (A/T and C/G) SNPs in EstBB genotype data were excluded. PRSs were calculated with PRS-cs[27], a Bayesian polygenic prediction method that places a continuous shrinkage prior on SNV effect sizes and infers posterior SNV weights using GWAS summary statistics restricted to ~1.1 million HapMap variants[28] and an external European sample-based LD reference from the 1000 Genomes Project[29]. The default parameters and the auto option were used, and the HLA region was excluded. For SCZ, however, the HLA region was considered and the global shrinkage parameter phi = 1 was used, given the significant association of the HLA region in SCZ GWAS and to capture the highly polygenic architecture of SCZ, respectively[18,30–33]. The PRSs were normalized to follow a normal distribution with mean of 0 and SD of 1.

Correlation patterns among 15 PRSs were assessed with Pearson correlation using R/*corrplot*[34]. Significant associations were identified based on Bonferroni correction (0.05/15 = 0.0033). To validate the correlation structure of the PRSs, unrelated EstBB participants (PLINK PI_HAT <0.2), excluding SCZ cases and controls used in the current study were used, totalling 117,792 individuals. Genetic correlations for 15 traits were calculated with LDSC[35], using summary statistics from GWAS restricted to SNVs in the HapMap 3 reference panel[28] and the LD scores from the European ancestry sample[29].

For testing the association with the derived median chlorpromazine-equivalent dose, PRSs for clozapine drug metabolites, i.e., clozapine, norclozapine and their ratio[36] were additionally included. Given that the PRSs built only with genome-wide significant variants showed stronger association and greater explained variance for these traits compared to those built using less stringent p-value thresholds[36], we considered only independent variants (--clump-r2 0.1) below $P<5\times10^{-8}$ and calculated the PRSs using PLINK (--score)[13,17].

Association testing and statistical analyses

Association testing between median chlorpromazine-equivalent dose and treatment variables

To test the association of the median chlorpromazine-equivalent dose with adherence ($PDC_{purchase}$ specifically, given its high correlation with other adherence variables) and treatment years over supply ($treatment\_years_{supply}$), linear regression was used with sex and birth year as covariates. Individuals whose median chlorpromazine-equivalent dose was greater than ±3SD from the mean on log10 scale were excluded (n = 6; Supplementary Figure 10B).

Association testing between PRSs with disease status and the median chlorpromazine-equivalent dose

For association testing with disease status, multivariate logistic regression analysis was performed in a forward stepwise manner. Namely, each PRS of interest was first modelled independently with the SCZ/control status as a dependent variable, and baseline characteristics, i.e., sex, birth year, and 10 genotype principal components (PCs), as covariates. Next, the PRSs were incrementally added to the main model, starting with the one with the lowest p-value and scanning through all other PRSs. The effective number of tests ($m_{eff;}$ R/*poolR*[37]) was applied to account for the PRS correlation structure (0.05/13 = 0.0038).

To test whether the median chlorpromazine-equivalent dose was associated with the genetic liability to MetS, multivariate linear regression analysis was applied similarly to the multivariate logistic regression model. Median dose on log10 scale as a dependent variable was regressed on 18 PRSs (including the PRSs for clozapine, norclozapine and their ratio) in a forward stepwise manner with $PDC_{purchase}$, $treatment\_years_{supply}$, sex, birth year and ten genotype PCs as covariates. Individuals whose median chlorpromazine-equivalent dose on log10 scale was greater than ±3SD from the mean were excluded (n = 6; Supplementary Figure 10B). Normality assumption for BMI was assessed with diagnostic plots of model residuals. Since indications of non-normality of the residuals were observed, Box-Cox transformation was used to determine the best possible transformation of the response variable. However, as the Box-Cox transformation did not result in perfect normality and the analysis with transformed response variable showed basically the same ANOVA p-values for effects of interest (<2e-16 for both models), we opted for using the model with untransformed BMI values for easier interpretability.

Survival analyses

To assess the difference in the incidence of MetS in SCZ cases compared to controls, multivariate Cox proportional hazards modelling was applied using R/*survival*[38]. The following disease endpoints were considered: type II diabetes (T2D, ICD-10 E11), hypercholesterolemia (ICD-10 E78), essential hypertension (ICD-10 I10), coronary heart disease (CHD, ICD-10 I20-I25), cardiovascular diseases (CVD, ICD-10 I20-I25, I61, I63, I64), and any metabolic disorder (the first occurrence of any of the outlined MetS endpoints). The study entry date for SCZ cases was the onset of SCZ, either first diagnosis or prescription, and the study entry date for controls was the date of SCZ onset for the matched SCZ case. The study end date was either the first occurrence of the diagnosis of the considered MetS endpoint, death, or the end of follow-up. All prevalent cases were excluded (i.e., SCZ cases and controls with the endpoint of interest before the study start date). Sex, BMI group, smoking status, PRSs, and 10 genotype PCs were considered as covariates. Bonferroni correction (0.05/6 = 0.0083) was applied to account for six endpoint-specific analyses. In each of the six endpoint-specific model, the PRSs were modelled in a forward stepwise manner and the effective number of tests was applied to account for the PRS correlation structure as described in the previous paragraph.

To assess the association between MetS and adherence captured within the first treatment year, multivariate Cox proportional hazards modelling was applied as described previously. Only SCZ cases who had dispensed antipsychotics at least a year (n = 480) were considered. The study entry date was one year after the first antipsychotic purchase.

BMI trajectory analyses

To test the difference of BMI trajectories between SCZ cases and controls, we employed linear mixed modelling (R/*lme4*[39], R/*lmerTest*[40]) considering that individuals had multiple BMI measurements, that BMI trajectory over time could be individual-

specific and accounting for non-linear age effects (as quartic polynomial) and the polygenic predisposition to BMI (BMI PRS). Population parameters (sex, smoking status (*ever* vs *never*), BMI PRS, 10 genotype PCs) were modelled as fixed effects and subject-specific effects (subject-specific random intercept and subject-specific age effect) were considered as random effects. The interaction term between BMI PRS and age at BMI measurement was additionally included to capture the age-dependent effect of BMI PRS. Firstly, we considered two models, a baseline model and a model that additionally included an interaction term between disease status and the age at BMI measurement (models outlined in Supplementary Table 5). Next, to account for the treatment effect, linear mixed modelling was similarly applied as in the first approach. We considered the model with the better fit identified in the first approach and compared it with a model where the quartic polynomial of treatment years was additionally included (models outlined in Supplementary Table 6). Model fit was assessed with ANOVA. In both analyses, 1,785 and 9,446 BMI measurements with on average 3.34 and 3.69 BMI measurements per individual were available for 532 SCZ cases and 2,519 controls, respectively.

To assess the impact of treatment factors, we focused on SCZ cases. We considered sex, age at BMI measurement, the PRS for BMI, smoking status (*ever* vs *never*), and 10 genotype PCs as fixed effects. Additionally, we used the median chlorpromazine-equivalent dose and adherence calculated based on purchase information two years prior to each BMI measurement and treatment years based on all supply information up to each BMI timepoint. Subject-specific effects (subject-specific random intercept and subject-specific age effect) were considered as random effects. The median chlorpromazine-equivalent dose was transformed to log10 scale. Timepoints with a single antipsychotic purchase preceding the BMI measurement within the two-year timeframe and individuals whose median dose on log10 scale deviated more than >3SD from the mean were excluded. In total, 745 BMI measurements were available for 282 SCZ cases with an average of 2.61 BMI measurements per individual. In all analyses, the BOBYQA method[41] for optimization was applied. BMI trajectories were derived using the *predict* function.

Normality assumption for BMI was assessed with diagnostic plots of the model residuals. As there were indications of non-normality of the residuals, Box-Cox transformation was used to determine the best possible transformation of the response variable. However, as the Box-Cox transformation did not achieve perfect normality and the analysis with transformed response variable showed basically the same ANOVA p-values for effects of interests (<2e-16 for both models), we opted for using the model using untransformed BMI values due to easier interpretability.

We have provided 83% confidence intervals for Figure 3. This confidence level was chosen to make comparisons between group means easier. Namely, Goldstein and Healy (1995) highlight that to indicate statistically significant differences (alpha = 0.05) between two means with non-overlapping confidence intervals, one should use 83% confidence intervals in graphs[42].

Statistical analyses were conducted with R software version 4.2.2.[43]

References for Supplementary Methods

1. Leitsalu L, Haller T, Esko T, et al. Cohort Profile: Estonian Biobank of the Estonian Genome Center, University of Tartu. *Int J Epidemiol* 2015; **44**: 1137–1147.
2. Leitsalu L, Alavere H, Tammesoo ML, Leego E, Metspalu A. Linking a Population Biobank with National Health Registries–The Estonian Experience. *J Pers Med* 2015; **5**: 96–106.
3. World Health Organization. 1993. The ICD-10 classification of mental and behavioural disorders: Diagnostic criteria for research. World Health Organization.
4. Ho D, Imai K, King G, Stuart EA. MatchIt: Nonparametric Preprocessing for Parametric Causal Inference. *J Stat Softw* 2011; **42**: 1–28.
5. Langlands RL, Jorm AF, Kelly CM, Kitchener BA. First Aid Recommendations for Psychosis: Using the Delphi Method to Gain Consensus Between Mental Health Consumers, Carers, and Clinicians. *Schizophr Bull* 2008; **34**; 435–443.
6. Hasson F, Keeney S, McKenna H. Research guidelines for the Delphi survey technique. *J Adv Nurs* 2000; **32**: 1008–1015.
7. Gardner DM, Murphy AL, O'Donnell H, Centorrino F, Baldessarini RJ. International Consensus Study of Antipsychotic Dosing. *Am J Psychiatry* 2010; **167**: 686–693.
8. Leucht S, Crippa A, Siafis S, Patel MX, Orsini N, Davis JM. Dose-Response Meta-Analysis of Antipsychotic Drugs for Acute Schizophrenia. *Am J Psychiatry* 2019; **177**: 342–353.
9. Leucht S, Samara M, Heres S, Davis JM. Dose Equivalents for Antipsychotic Drugs: The DDD Method. *Schizophr Bull* 2016; **42**: S90–S94.
10. Webpage: https://github.com/ropensci/chlorpromazineR. Accessed in January 2024.
11. Webpage: https://www.whocc.no/ddd/definition_and_general_considera/. Accessed in January 2024.
12. Barnes TRE, Drake R, Paton C, et al. Evidence-based guidelines for the pharmacological treatment of schizophrenia: Updated recommendations from the British Association for Psychopharmacology. *J Psychopharmacol* 2020; **34**: 3–78.

13. Chang CC, Chow CC, Tellier TC, Vattikuti S, Purcell SM, Lee JJ. Second-generation PLINK: rising to the challenge of larger and richer datasets. *Gigascience* 2015; **4**: s13742-015-0047–8.
14. Loh PR, Danecek P, Palamara PF, et al. Reference-based phasing using the Haplotype Reference Consortium panel. *Nat Genet* 2016; **48**: 1443–1448.
15. Browning SR, Browning BL. Rapid and accurate haplotype phasing and missing-data inference for whole-genome association studies by use of localized haplotype clustering. *Am J Hum Genet* 2007; **81**: 1084–1097.
16. Mitt M, Kals M, Pärn K, et al. Improved imputation accuracy of rare and low-frequency variants using population-specific high-coverage WGS-based imputation reference panel. *Eur J Hum Genet* 2017; **25**: 869.
17. Purcell, S, Neale B, Todd-Brown K, et al. PLINK: A Tool Set for Whole-Genome Association and Population-Based Linkage Analyses. *Am J Hum Genet* 2007; **81**: 559–575.
18. Trubetskoy V, Pardiñas AF, Qi T, et al. Mapping genomic loci implicates genes and synaptic biology in schizophrenia. *Nature* 2022; **604**: 502–508.
19. Aragam KG, Jiang T, Goel A, et al. Discovery and systematic characterization of risk variants and genes for coronary artery disease in over a million participants. *Nat Genet* 2022; **54**: 1803–1815.
20. Graham SE, Clarke SL, Wu KHH, et al. The power of genetic diversity in genome-wide association studies of lipids. *Nature* 2021; **600**: 675–679.
21. Said S, Pazoki R, Karhunen V, et al. Genetic analysis of over half a million people characterises C-reactive protein loci. *Nat Commun* 2022; **13**: 2198.
22. Chen J, Spracklen CN, Marenne G, et al. The trans-ancestral genomic architecture of glycemic traits. *Nat Genet* 2021; **53**: 840–860.
23. Lagou V, Jiang L, Ulrich A, et al. GWAS of random glucose in 476,326 individuals provide insights into diabetes pathophysiology, complications and treatment stratification. *Nat Genet* 2023; **55**: 1448–1461.
24. Mahajan A, Spracklen CN, Zhang W, et al. Multi-ancestry genetic study of type 2 diabetes highlights the power of diverse populations for discovery and translation. *Nat Genet* 2022; **54**: 560–572.
25. Yengo L, Sidorenko J, Kemper KE, et al. Meta-analysis of genome-wide association studies for height and body mass index in ~700000 individuals of European ancestry. *Hum Mol Genet* 2018; **27**: 3641–3649.
26. Evangelou E, Warren HR, Mosen-Ansorena D, et al. Genetic analysis of over 1 million people identifies 535 new loci associated with blood pressure traits. *Nat Genet* 2018; **50**: 1412–1425.
27. Ge T, Chen CY, Ni Y, Feng YCA, Smoller JW. Polygenic prediction via Bayesian regression and continuous shrinkage priors. *Nat Commun* 2019; **10**: 1776.
28. The International Hapmap 3 Consortium. Integrating common and rare genetic variation in diverse human populations. *Nature* 2010; **467**, 52.
29. 1000 Genomes Project Consortium, Auton A, Brooks LD, et al. A global reference for human genetic variation. *Nature* 2015; **526**: 68–74.
30. Kappel DB, Legge SE, Hubbard L, et al. Genomic Stratification of Clozapine Prescription Patterns Using Schizophrenia Polygenic Scores. *Biol Psychiatry* 2023; **93**: 149–156.
31. Holland D, Frei O, Desikan R, et al. Beyond SNP heritability: Polygenicity and discoverability of phenotypes estimated with a univariate Gaussian mixture model. *PLoS Genet* 2020; **16**: e1008612.
32. Alver M, Mancini V, Läll K, et al. Contribution of schizophrenia polygenic burden to longitudinal phenotypic variance in 22q11.2 deletion syndrome. *Mol Psychiatry* 2022; **27**: 4191–4200.
33. Zheutlin AB, Dennis J, Linnér RK, et al. Penetrance and Pleiotropy of Polygenic Risk Scores for Schizophrenia in 106,160 Patients Across Four Health Care Systems. *Am J Psychiatry* 2019; **176**: 846–855.
34. Wei T, Simko V. R package 'corrplot': Visualization of a Correlation Matrix. 2020. Available from https://github.com/taiyun/corrplot.
35. Bulik-Sullivan B, Finucane HK, Anttila V, et al. An atlas of genetic correlations across human diseases and traits. *Nat Genet* 2015; **47**: 1236–1241.
36. Pardiñas AF, Kappel DB, Roberts M, et al. Pharmacokinetics and pharmacogenomics of clozapine in an ancestrally diverse sample: a longitudinal analysis and genome-wide association study using UK clinical monitoring data. *Lancet Psychiatry* 2023; **10**: 209–219.
37. Cinar O, Viechtbauer W. The poolr Package for Combining Independent and Dependent p Values. *J Stat Softw* 2022; **101**: 1–42.
38. Therneau T. A Package for Survival Analysis in R. 2023 Available from https://CRAN.R-project.org/package=survival.
39. Bates D, Mächler M, Bolker B, Walker S. Fitting linear mixed-effects models using lme4. *J Stat Softw* 2015; **67**: 1–48.
40. Kuznetsova A, Brockhoff PB, Christensen RHB. lmerTest Package: Tests in Linear Mixed Effects Models. *J Stat Softw* 2017; **82**: 1–26.
41. Powell MJD. The BOBYQA algorithm for bound constrained optimization without derivatives. DAMTP 2009/NA06, Centre for Mathematical Sciences, University of Cambridge, UK (August 2009).
42. Goldstein H, Healy MJR. The Graphical Presentation of a Collection of Means. *Journal of the Royal Statistical Society* 1995; **158**: 175–177.
43. R Core Team. R: A Language and Environment for Statistical Computing. R Foundation for Statistical Computing. 2022. Vienna, Austria.

SUPPLEMENTARY FIGURES

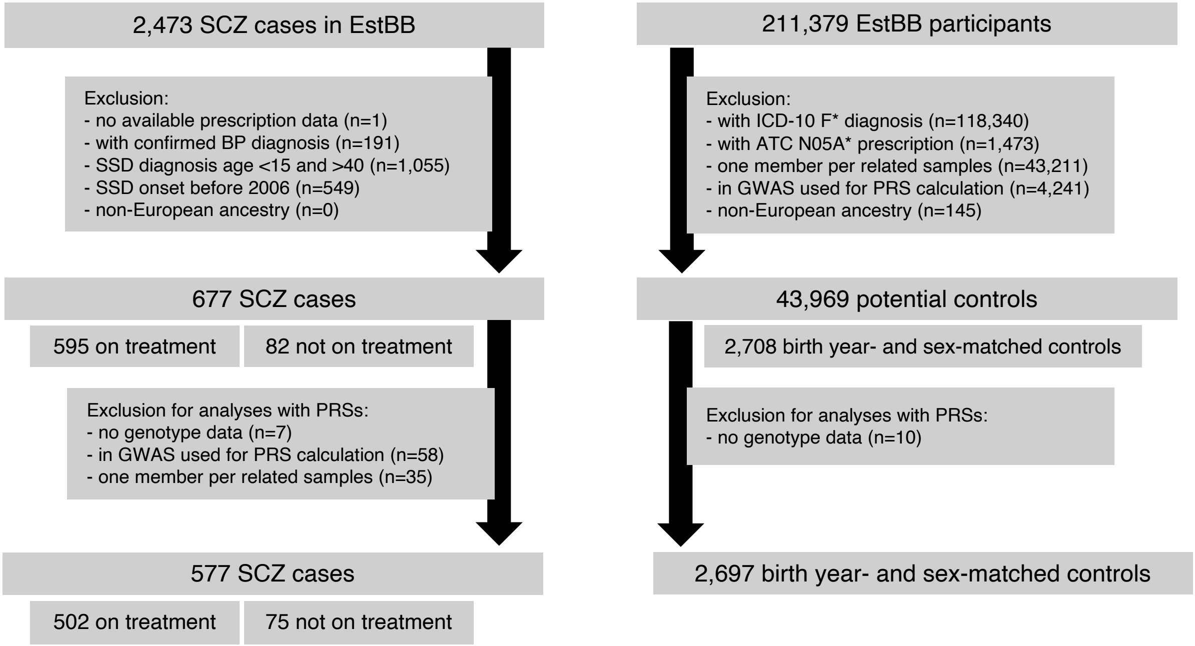

Supplementary Figure 1. Flowchart for selecting SCZ cases and birth year- and sex-matched controls in EstBB. The first section outlines the exclusion criteria for defining SCZ cases and controls for the study. The second section outlines the exclusion criteria for deriving the sample sets for analyses with PRSs.

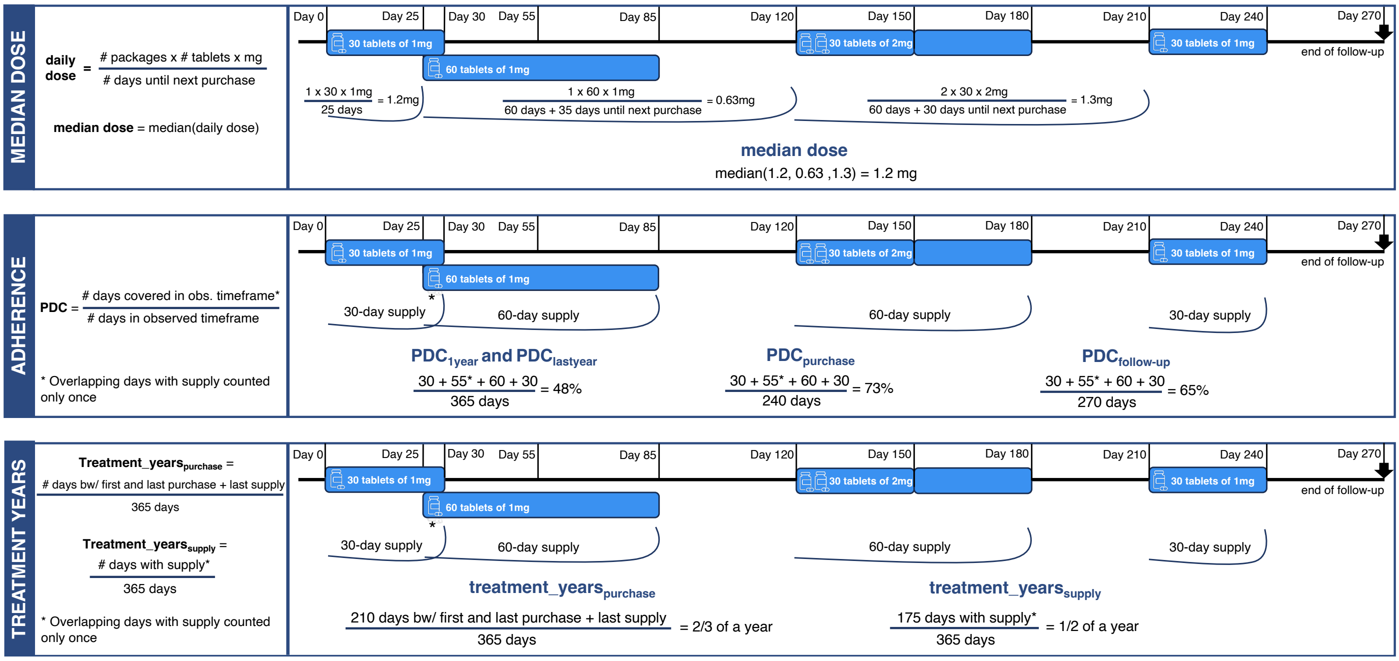

Supplementary Figure 2. Overview of the derivation of treatment variables from digital drug dispensing data. In each panel, the top part displays four purchases (1 package of 30 tablets of 1mg, 1 package of 60 tablets of 1mg, 2 packages of 30 tablets of 2mg, and 1 package of 30 tablets of 1mg) for an individual over a 270-day period. A shorter time period (<1 year) is considered for simplicity. The half balloons below each purchase in each section indicate the values used for the derivation of the treatment variables. For each treatment variable, one pill per day is considered. Of note, the second purchase of 1 package of 60 tablets of 1mg is depicted to occur 5 days before the end of the supply of the first purchase.

Median dose: For each purchase, the package content (i.e., dose in mg and the number of pills in a package) was multiplied by the number of packages bought. The calculated *mg × pills in a package × number of packages* variable for each purchase was divided by the number of days until the next purchase resulting in an estimate of a daily dose per purchase. The median daily antipsychotic dose was derived by taking the median across the derived daily doses.

Adherence: The proportion of days covered within the first treatment year (PDC$_{1year}$) was calculated by counting the days with antipsychotic supply for all purchased antipsychotics within a year from the first purchase and divided by 365 days. The proportion of days covered within the last available treatment year (PDC$_{lastyear}$) was calculated by determining the days' supply for all purchased antipsychotics within their last available treatment year divided by 365 days. The proportion of days covered over the dispensing period (PDC$_{purchase}$) was derived by calculating days' supply for all purchased antipsychotics from the first purchase until the end of supply of the last purchase and divided by the number of days between the first purchase and the end of supply. The proportion of days covered over the study period (PDC$_{follow\text{-}up}$) was derived by calculating the days' supply for all purchased antipsychotics from the first purchase until the end of follow-up divided by the number of days between the first purchase and the end of follow-up. For all adherence variables, the days' supply was calculated by multiplying the number of pills in a package by the number of packages bought per purchase, assuming one pill per day. Overlapping days with supply were counted only once as indicated by an asterisk.

Treatment years: To derive treatment_years$_{purchase}$, we counted the days from the first to the last purchase, added the supply of the last purchase and converted the summed days to a year. For treatment_years$_{supply}$, we added up the days' supply for each purchase and converted the number to a year. Overlapping days with supply were counted only once as indicated by an asterisk.

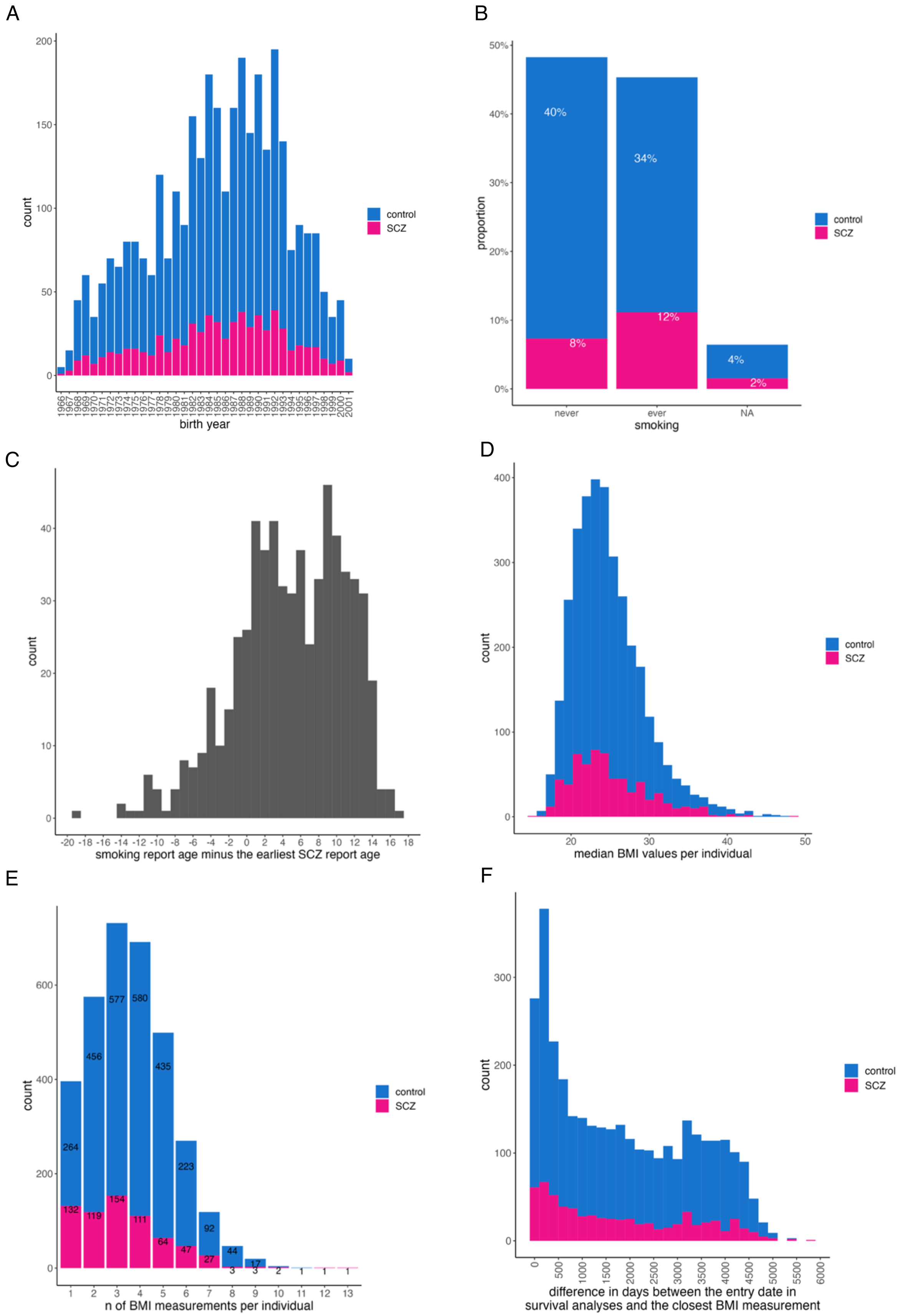

Supplementary Figure 3. Smoking and BMI data. (A) Overview of the distribution of birth years for SCZ cases and controls coloured for disease status. (B) Proportion of individuals who reported ever/never smoking. (C) For SCZ cases, the difference between the age when smoking was reported in EstBB and the age at SCZ onset (either first prescription or first diagnosis) based on electronic health records. The age at SCZ onset is subtracted from the age when smoking was reported in EstBB on x-axis. (D) Distribution of the median BMI measurement values per individual. (E) Count of SCZ cases and controls by the number of BMI measurements over follow-up. (F) Difference in days between the study entry date in survival analyses (i.e., date of SCZ onset for SCZ cases and matched controls).

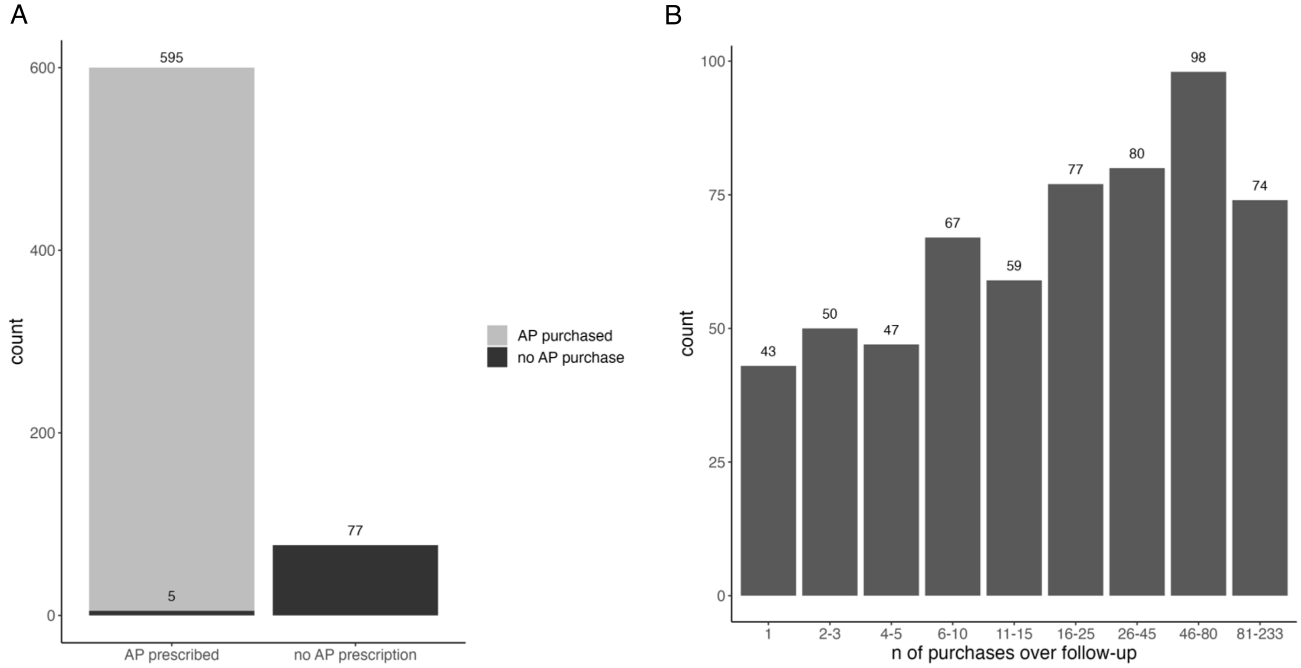

Supplementary Figure 4. Distributions of antipsychotic purchases. (A) Number of SCZ cases who were prescribed and who purchased antipsychotics (AP). (B) Count of SCZ cases by the number of antipsychotic purchases over follow-up.

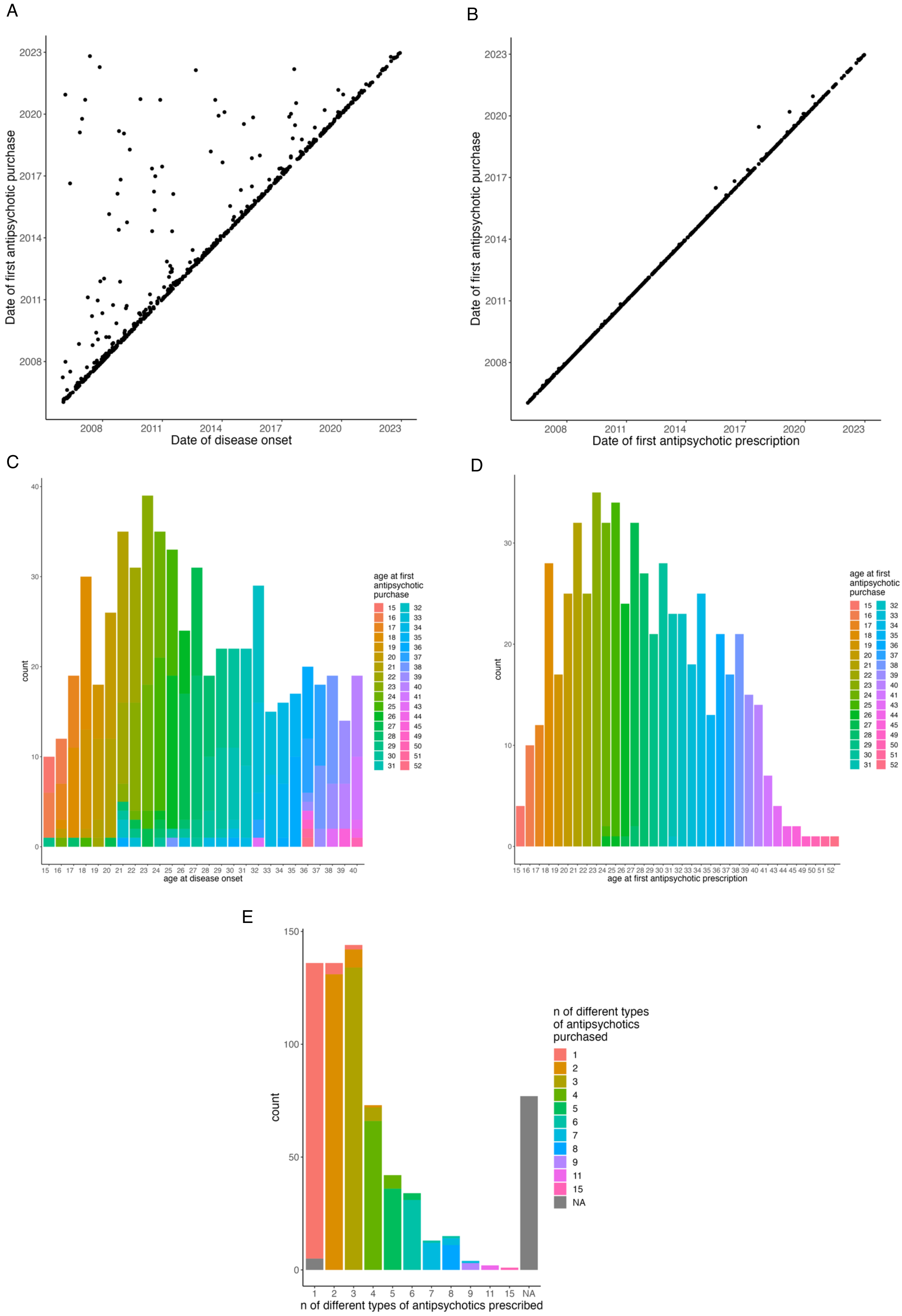
A
Date of first antipsychotic purchase
Date of disease onset
2008
2011
2014
2017
2020
2023
B
Date of first antipsychotic purchase
Date of first antipsychotic prescription
C
count
age at disease onset
age at first antipsychotic purchase
D
count
age at first antipsychotic prescription
age at first antipsychotic purchase
E
count
n of different types of antipsychotics prescribed
n of different types of antipsychotics purchased
NA

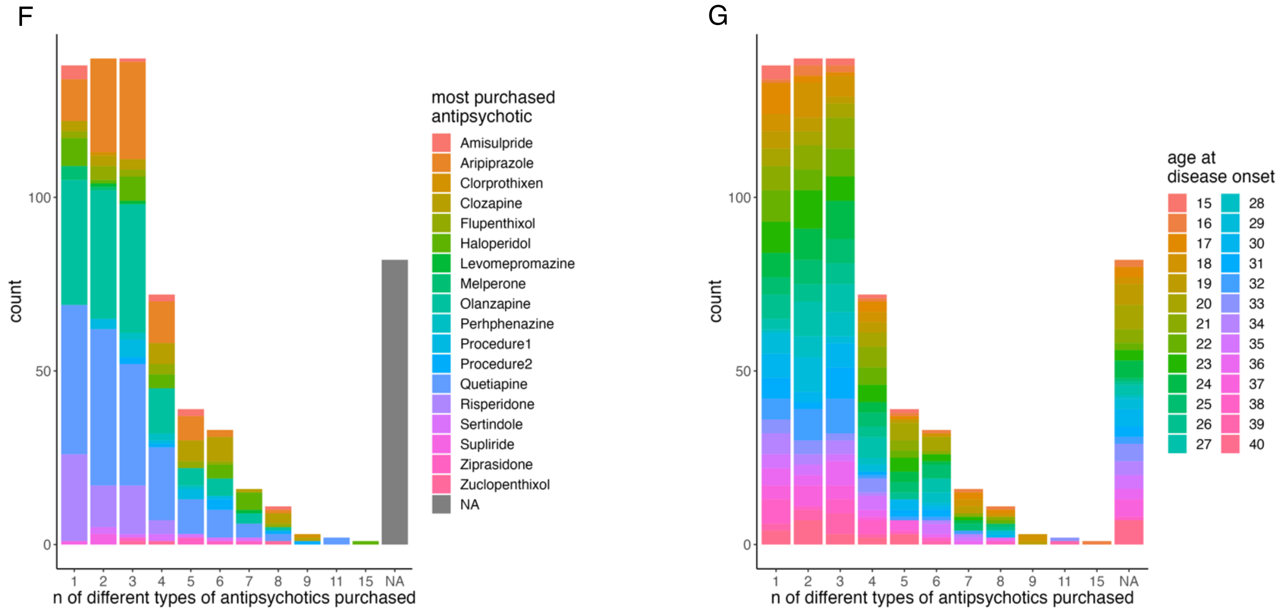

Supplementary Figure 5. Overview of antipsychotic purchases. (A) Date of disease onset (either first disease diagnosis or first antipsychotic prescription) as a function of the date of the first antipsychotic purchase. (B) Date of the first antipsychotic prescription as a function of the date of the first antipsychotic purchase. (C) Overview of the age at disease onset coloured by the age at first antipsychotic purchase. (D) Overview of the age at first antipsychotic prescription coloured by the age at first antipsychotic purchase. (E) Number of different types of antipsychotics prescribed per SCZ case coloured by the number of different types of antipsychotics purchased per SCZ case over follow-up. NA indicates antipsychotics either not prescribed or not purchased. (F). Number of different types of antipsychotics purchased per SCZ case coloured by the most purchased antipsychotic per SCZ case. Procedure1 indicates outpatient procedures for long-acting injections of Risperidone, Olanzapine, Aripiprazole, Paliperidone provided by a psychiatric nurse, Procedure2 indicates outpatient procedures for long-acting injections of Perphenazine, Fluphenazine provided by a psychiatric nurse. NA indicates antipsychotics not purchased. (G) Number of different types of antipsychotics purchased per SCZ case coloured by the age at disease onset. NA indicates antipsychotic not purchased.

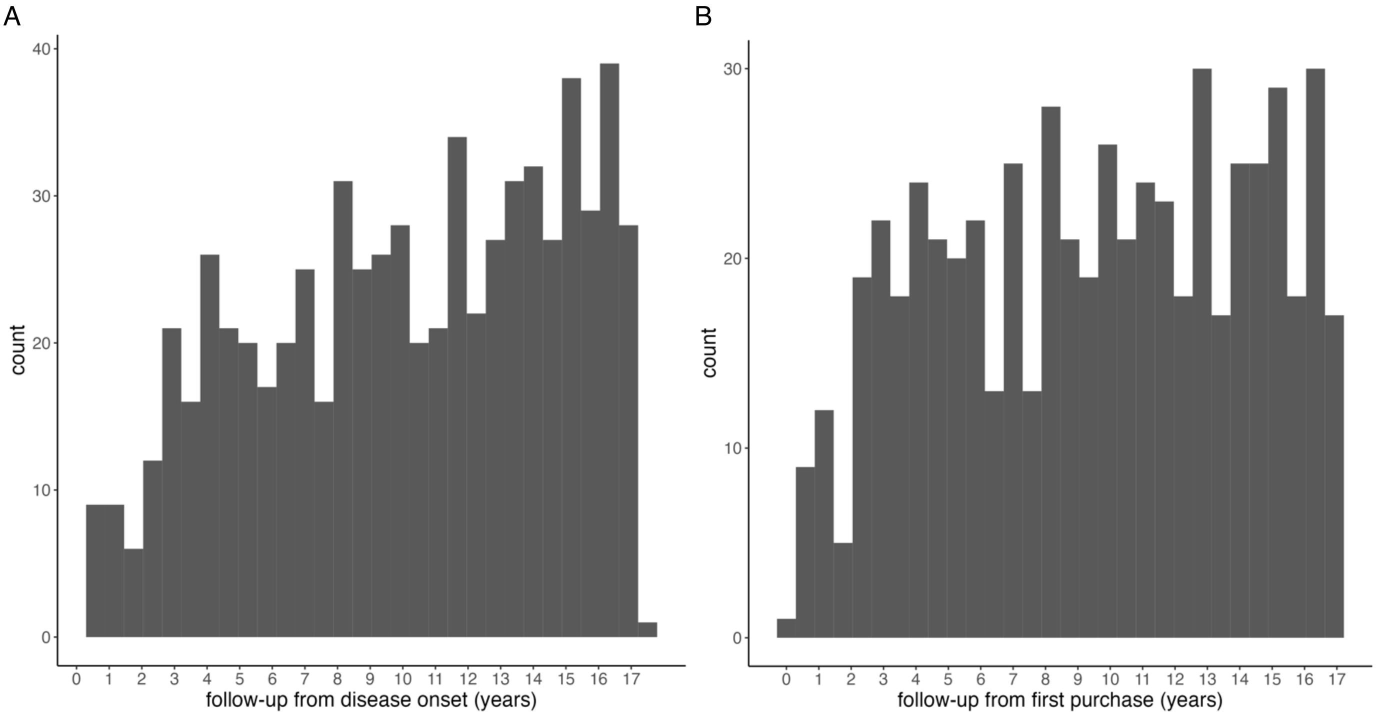

Supplementary Figure 6. Follow-up of SCZ cases. (A) Distribution of the follow-up period in years from disease onset (either first diagnosis or first antipsychotic prescription). (B) Distribution of the follow-up period in years from the first antipsychotic purchase.

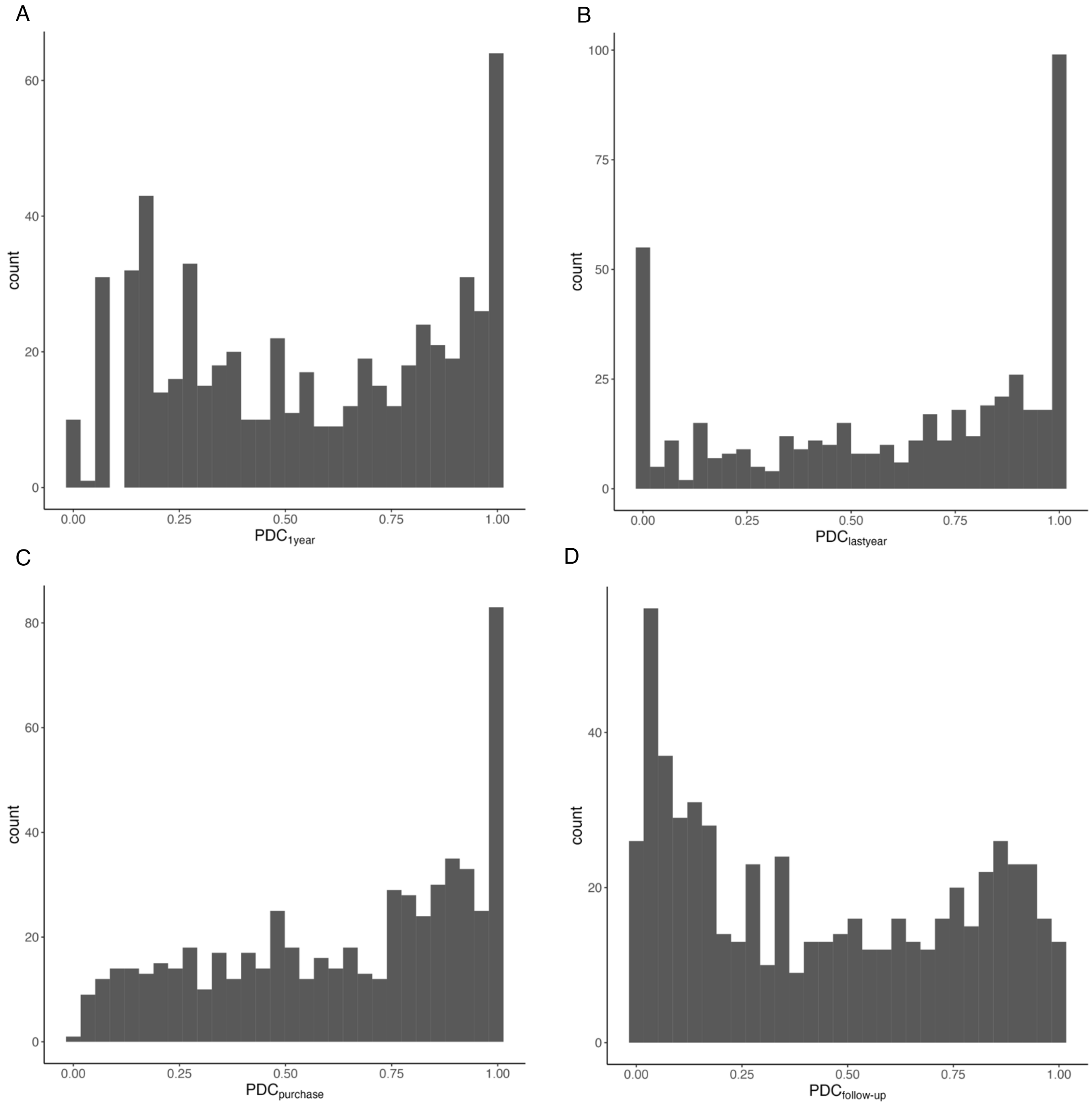

Supplementary Figure 7. Distributions of adherence variables. (A) PDC_{1year}, (B) $PDC_{lastyear}$, (C) $PDC_{purchase}$, (D) $PDC_{follow\text{-}up}$.

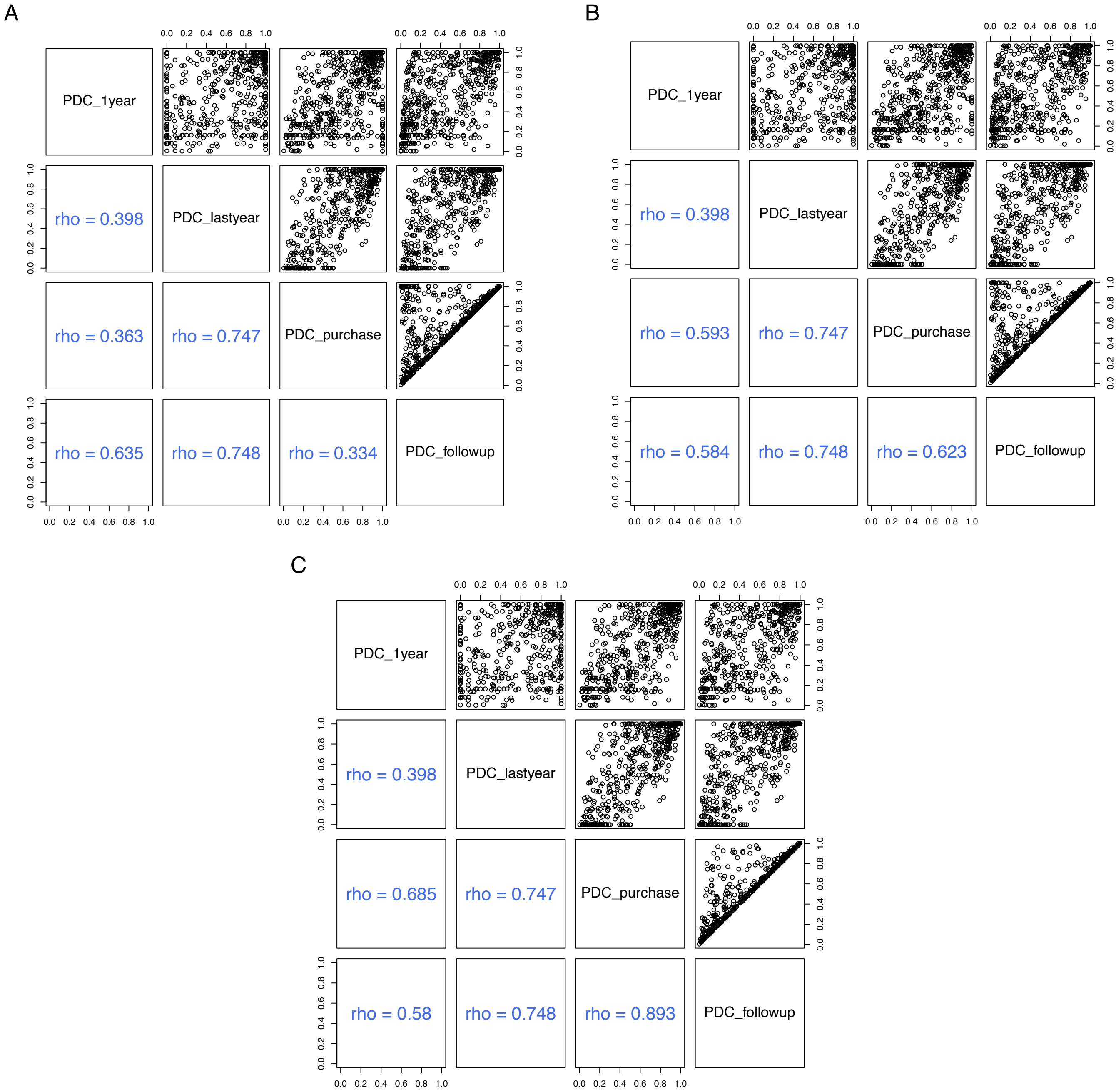

Supplementary Figure 8. Spearman correlations among adherence variables. The Spearman correlations were derived (A) for all SCZ cases (n = 595), (B) among SCZ with >1 purchase (n = 550), (C) among SCZ cases with at least one year between the first and the last antipsychotic purchase (n = 480). Of note, PDC$_{1year}$ was derived for SCZ cases whose first antipsychotic prescription was dispensed at least one year before the end of follow-up (n = 582). PDC$_{lastyear}$ was derived for SCZ cases who had purchase information available for more than one year (n = 480). PDC$_{follow\text{-}up}$ and PDC$_{purchase}$ was derived for all SCZ cases who had purchased antipsychotics at least once (n = 595).

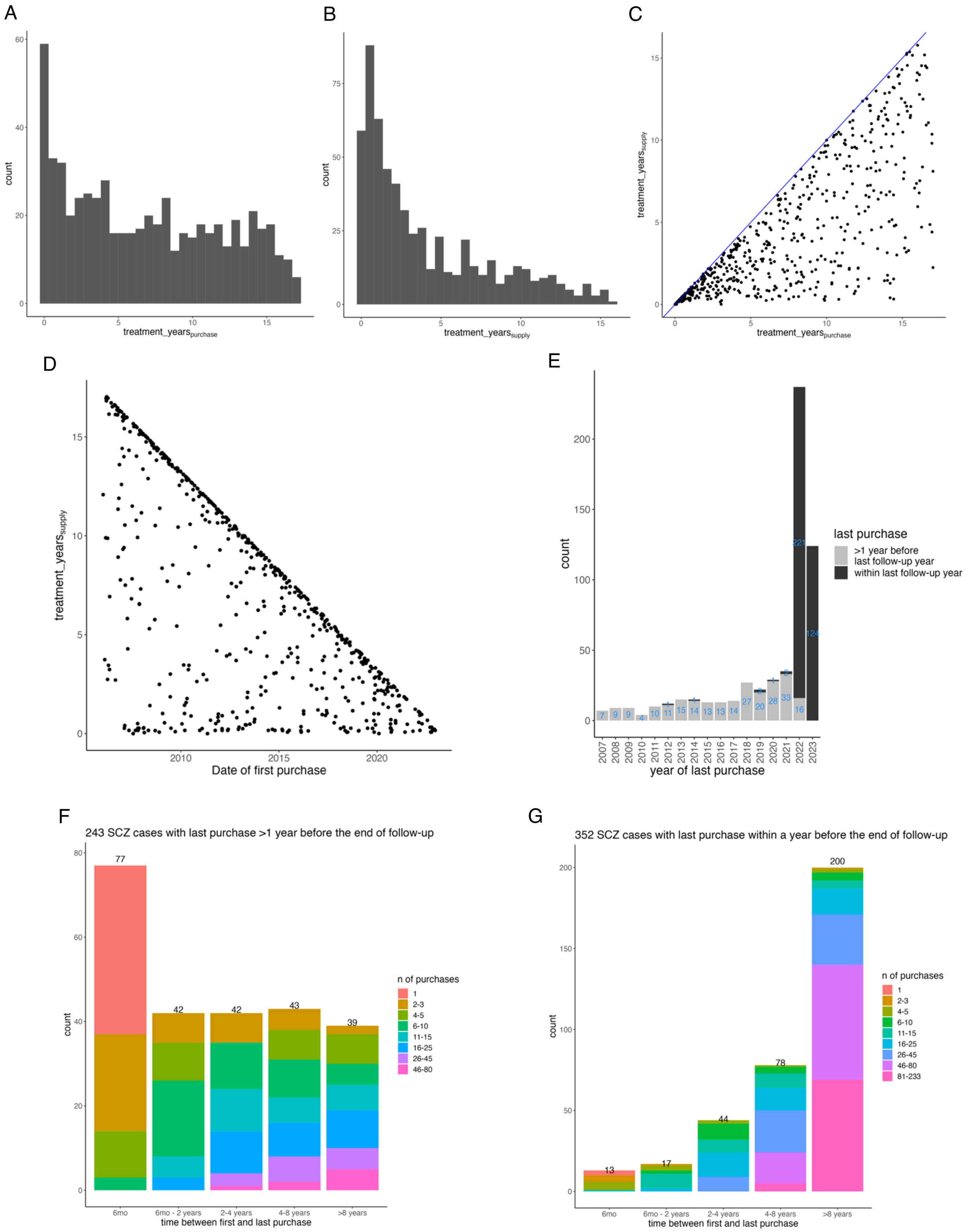
A
B
C
D
E
F
G
count
treatment_years_purchase
treatment_years_supply
Date of first purchase
year of last purchase
last purchase
>1 year before last follow-up year
within last follow-up year
243 SCZ cases with last purchase >1 year before the end of follow-up
352 SCZ cases with last purchase within a year before the end of follow-up
n of purchases
time between first and last purchase
6mo
6mo - 2 years
2-4 years
4-8 years
>8 years

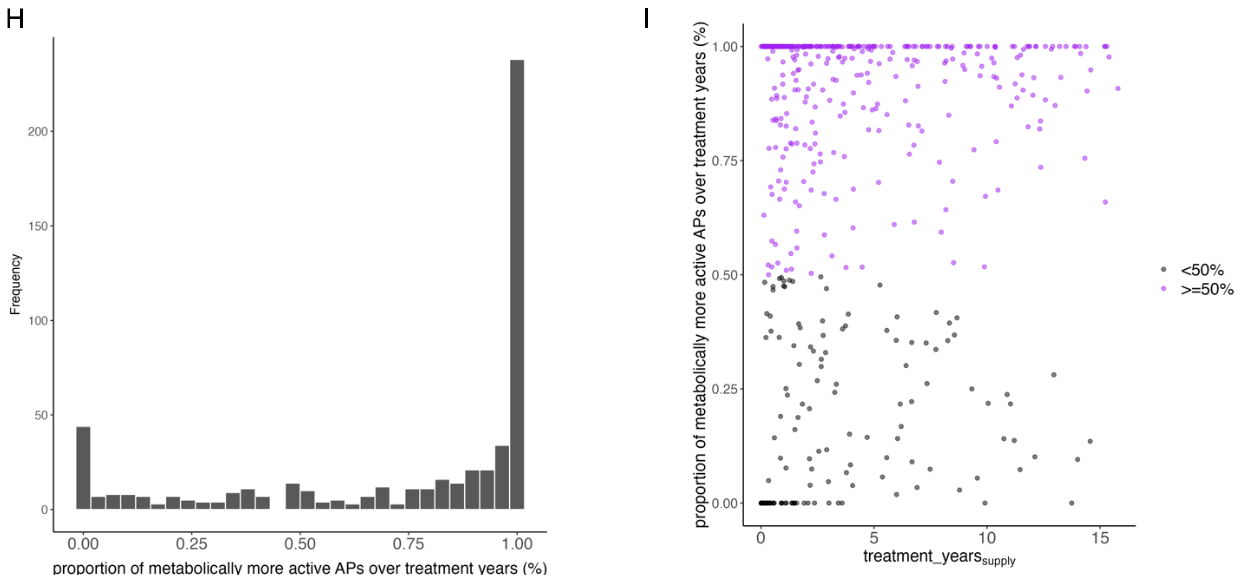

Supplementary Figure 9. Treatment years variables. (A) Distribution of the length of treatment from first to last purchase in years. (B) Distribution of the length of treatment based on days with antipsychotic supply. (C) Correlation between treatment length variables with the length of treatment from first to last purchase on x-axis and the length of treatment based on days with antipsychotic supply on y-axis. (D) Overview of treatment length from first to last purchase by the date of the first purchase. (E) Overview of the number of SCZ cases by the year the last antipsychotics were purchased. For 13 individuals who died before the end of follow-up, the last year antipsychotics were purchased was calculated based on the date of death. (F) Overview of the time between the first and the last purchase coloured by the number of purchases for 243 SCZ cases with last purchase more than one year before the end of follow-up/death. (G) Overview of the time between the first and the last purchase coloured by the number of purchases for 352 SCZ cases with the last purchase within a year before the end of follow-up/death. (H) Distribution of the proportion of metabolically more active antipsychotics (APs) over treatment years based on days with APs supply ($treatment\_years_{supply}$). Clozapine, risperidone, quetiapine, and olanzapine were considered as metabolically more active APs. (I) Proportion of metabolically more active APs over treatment years (%) in relation to treatment years based on days with AP supply ($treatment\_years_{supply}$). Individuals who purchased metabolically more active APs >=50% of their treatment years are coloured purple.

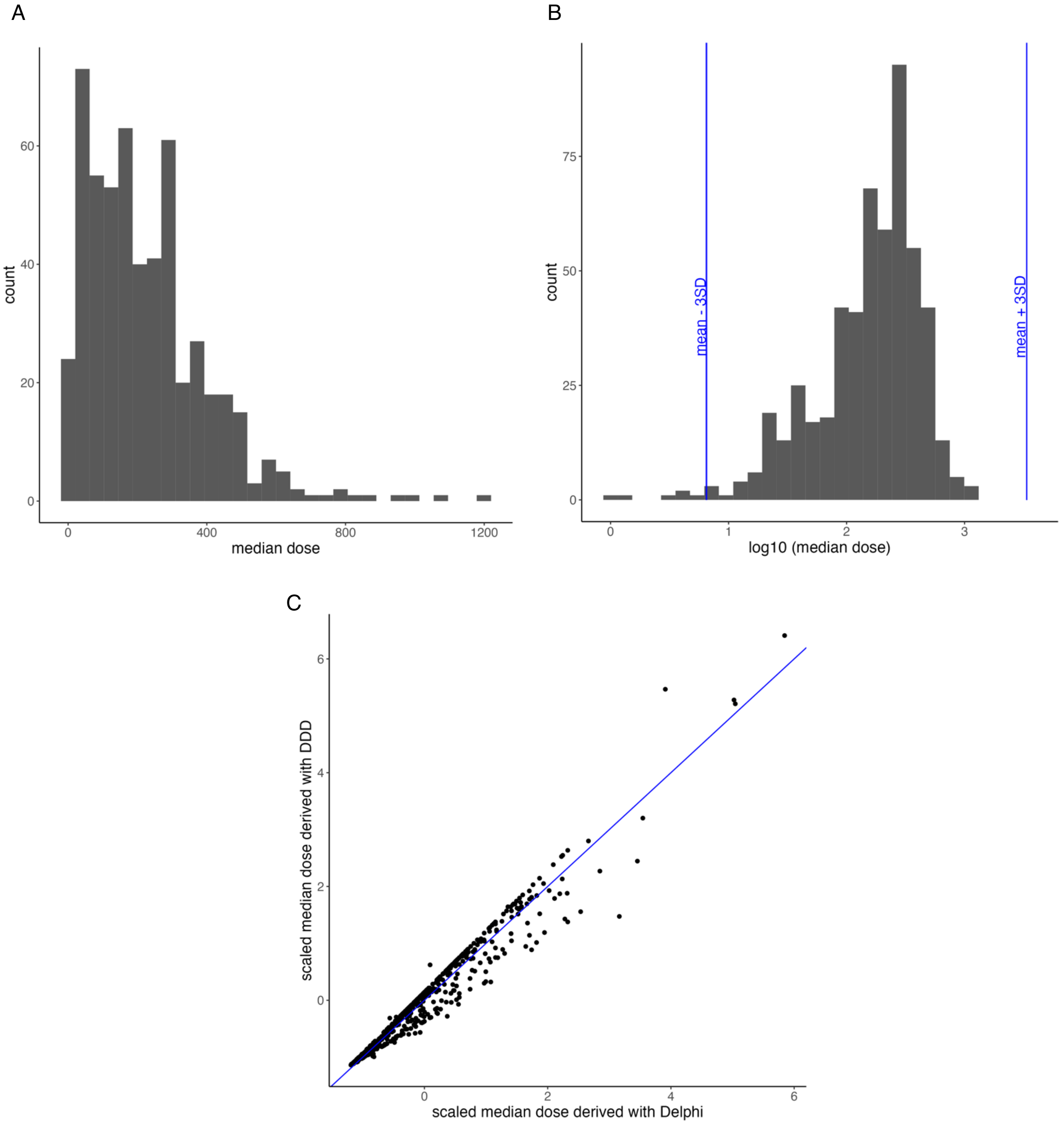

Supplementary Figure 10. Median dose. (A) Distribution of the median chlorpromazine-equivalent dose values in mg for SCZ cases. (B) Distribution of the median chlorpromazine-equivalent dose values on log10 scale with blue lines indicating ±3SD from mean. (C) Pearson correlation between the median dose derived with the Delphi method vs the median dose derived with the Defined Daily Dose (DDD) method. The derived median doses are standardized such that these follow a normal distribution with mean 0 and SD 1 due to differences in distribution (the Delphi-derived median doses vary from 0 to 1,250 and the DDD-derived median doses from 0 to 4).

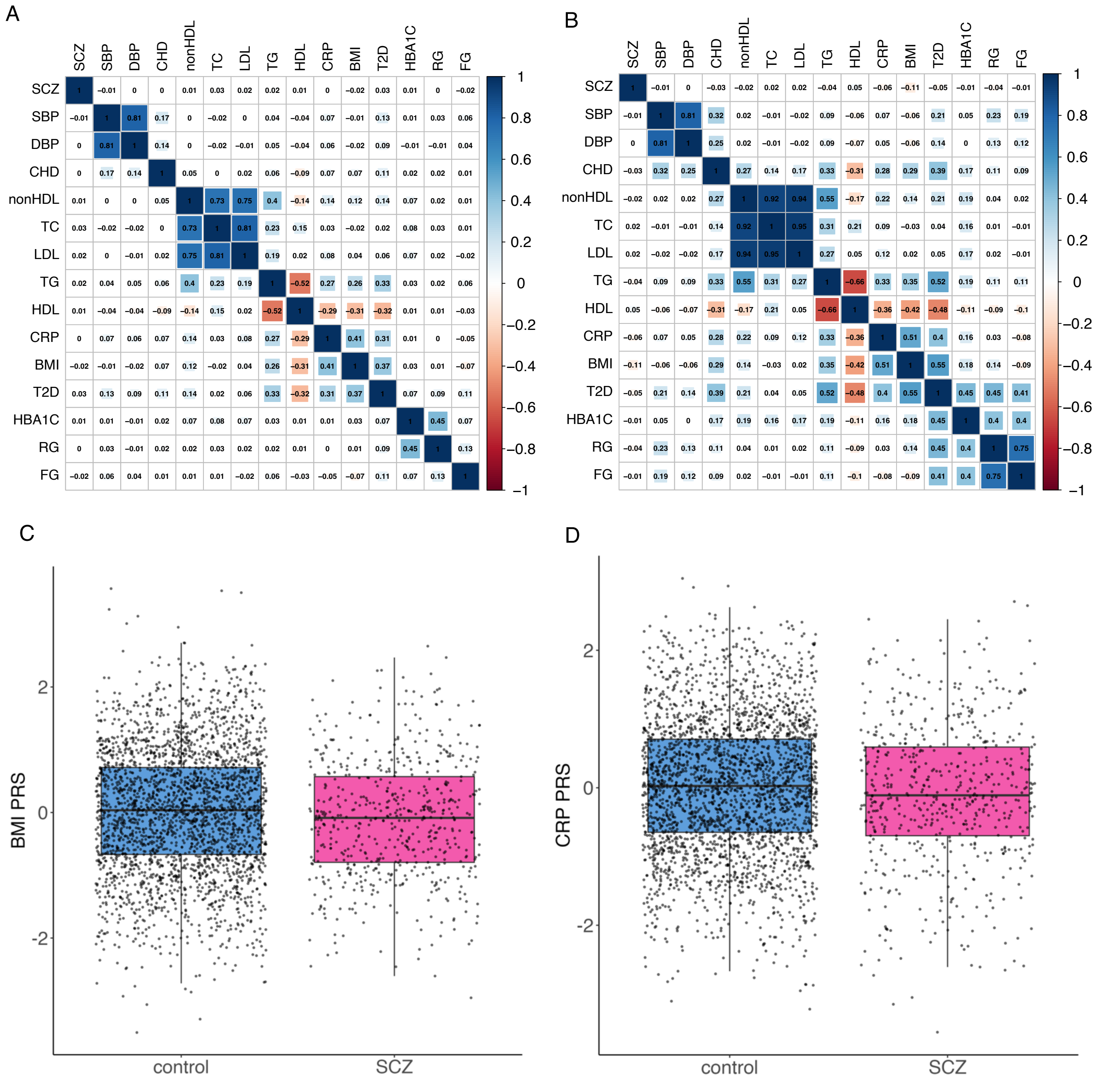

Supplementary Figure 11. PRSs of MetS traits and SCZ. (A) Correlation matrix of the PRSs for 14 MetS traits and SCZ using unrelated EstBB participants (n = 117,792). (B) Genetic correlations retrieved with LDSC based on published GWAS for 14 MetS traits and SCZ. (C) Boxplots of BMI PRS distribution for SCZ cases and controls. (D) Boxplots of CRP PRS distribution for SCZ cases and controls.

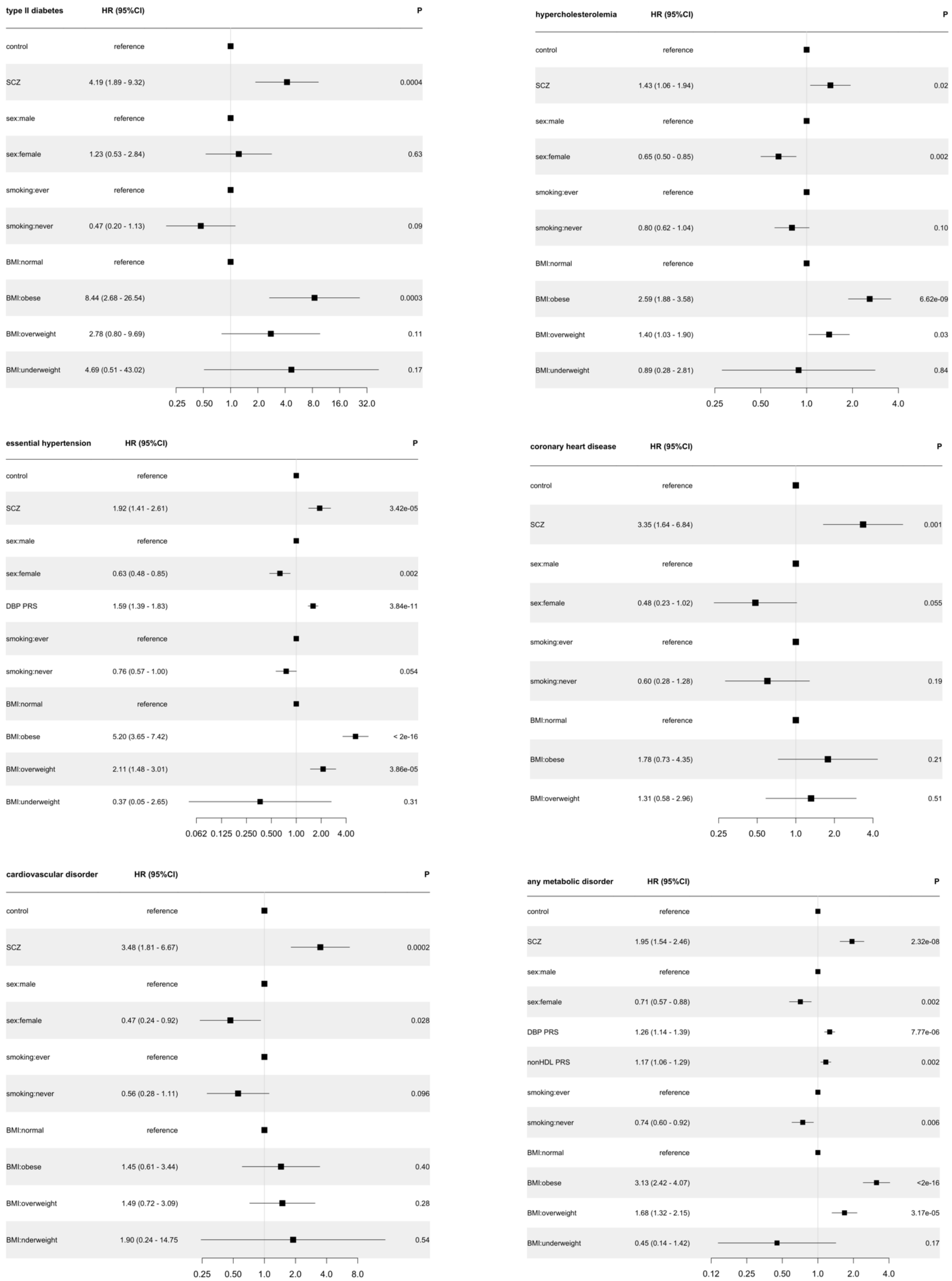

Supplementary Figure 12. Forest plots of endpoint-specific survival analyses. Genotype PCs are omitted for clarity.

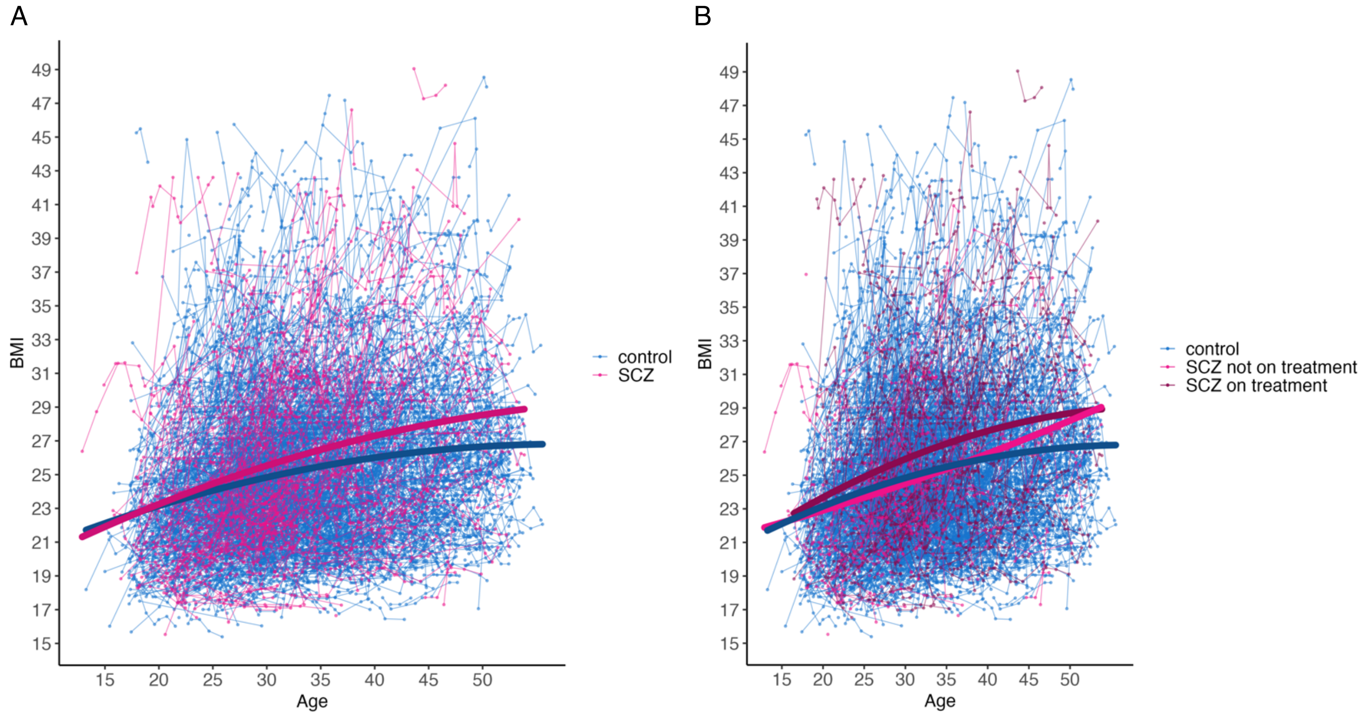

Supplementary Figure 13. Distribution of raw BMI measurement values. These are outlined across time (A) for SCZ cases and controls, and (B) for SCZ cases on treatment, SCZ not on treatment, and controls. Each dot denotes a BMI measurement determined at given timepoint connected by a straight line for each individual.

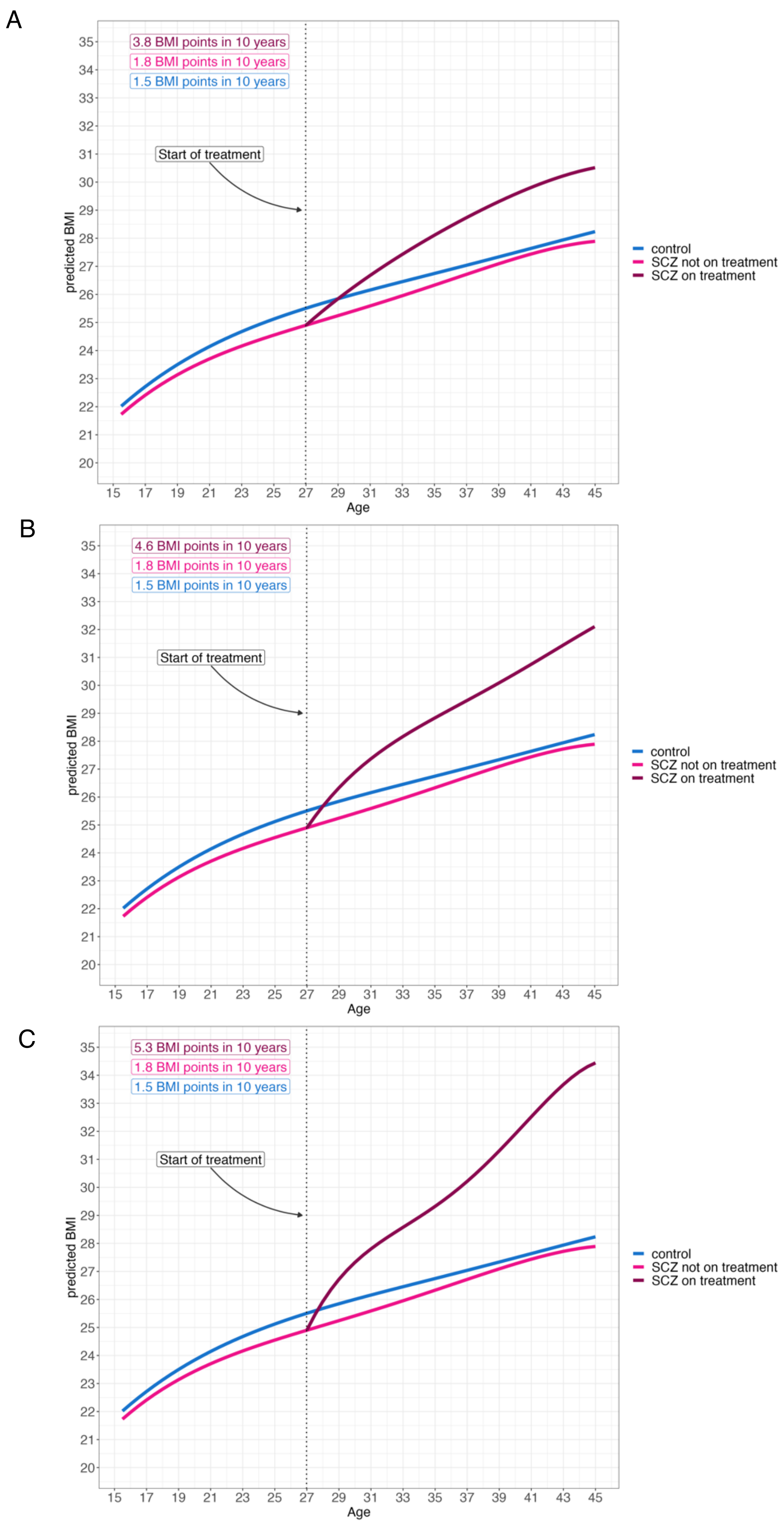

Supplementary Figure 14. Predicted BMI trajectories over time modelled for a SCZ case using different levels of antipsychotic supply per year in relation to a SCZ case not on treatment and a control individual. The BMI trajectories for a SCZ taking antipsychotics from age 27 (A) 1/4 of a year, (B) 1/2 a year, (C) 3/4 of a year. An increase in BMI over a decade is outlined for a SCZ case on treatment (dark pink), for a SCZ case not on treatment (pink) and for a control (blue) considering a male individual with BMI PRS equal to the population average and with smoking history. The black dotted line indicates the age at treatment initiation for a SCZ case.

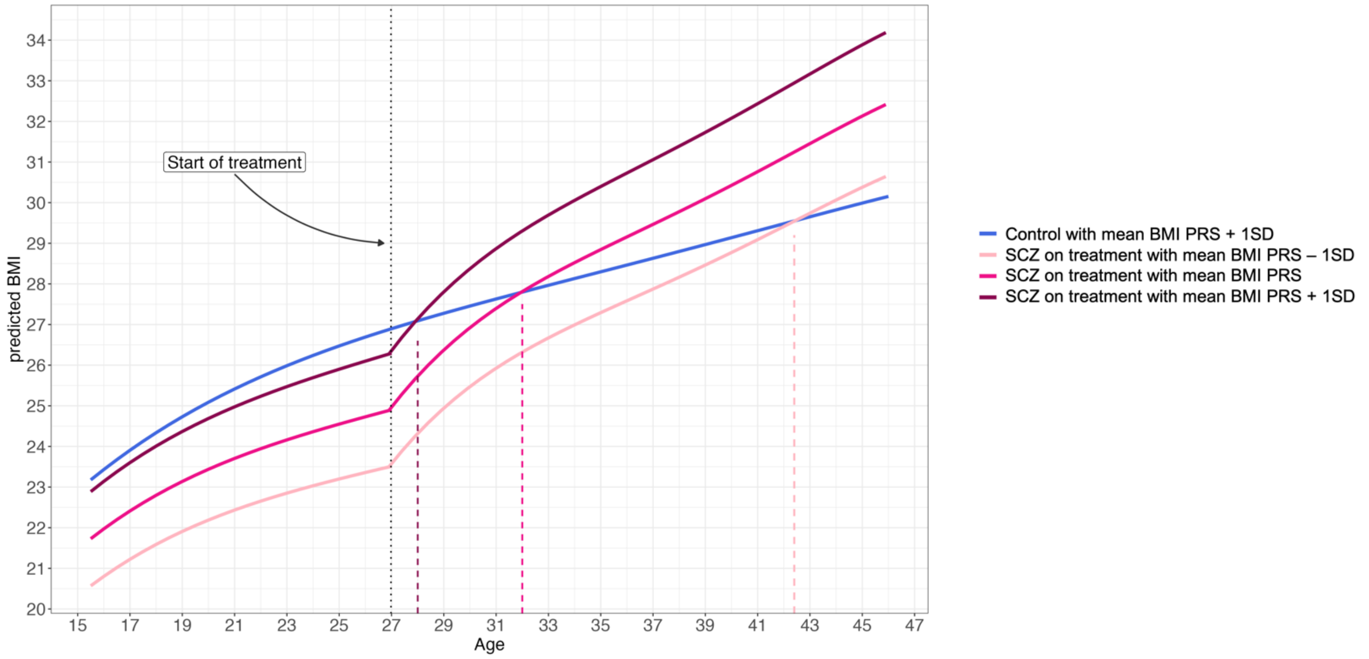

Supplementary Figure 15. Predicted BMI trajectories over time for control with high BMI PRS and a SCZ case with different BMI PRS values. The BMI trajectories are indicated for a male control with BMI PRS that is 1SD higher than the population average (blue), for a male SCZ case on treatment with BMI PRS that is 1SD lower than the population average (light pink), for a male SCZ case on treatment with BMI PRS that equals to the population average (pink), and for a male SCZ case on treatment with BMI PRS that is 1SD higher than the population average (dark pink). The coloured dashed lines indicate the age when the SCZ reaches the same level of BMI as the control with BMI PRS that is 1SD higher than the population average. The black dotted line indicates the age at treatment initiation for a SCZ case (age 27). Treatment is considered as taking antipsychotics 180 days every year. All individuals are considered to have a history of smoking.

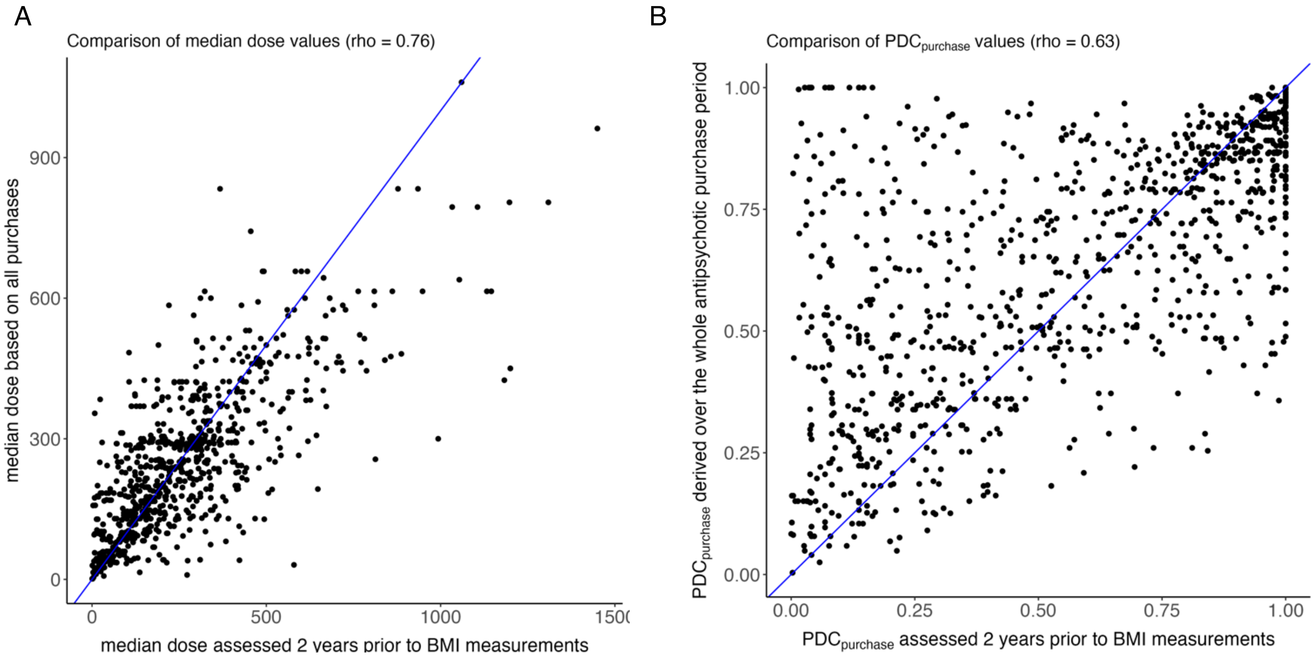

Supplementary Figure 16. Comparison of median dose and adherence values derived using different time scales. (A) Spearman correlation between the median chlorpromazine-equivalent dose assessed two years prior to each BMI measurement (x-axis) vs the median chlorpromazine-equivalent dose assessed using all purchase data (y-axis). (B) Spearman correlation between $PDC_{purchase}$ assessed two years prior to each BMI measurement (x-axis) vs $PDC_{purchase}$ derived over the whole antipsychotic purchase period (y-axis).

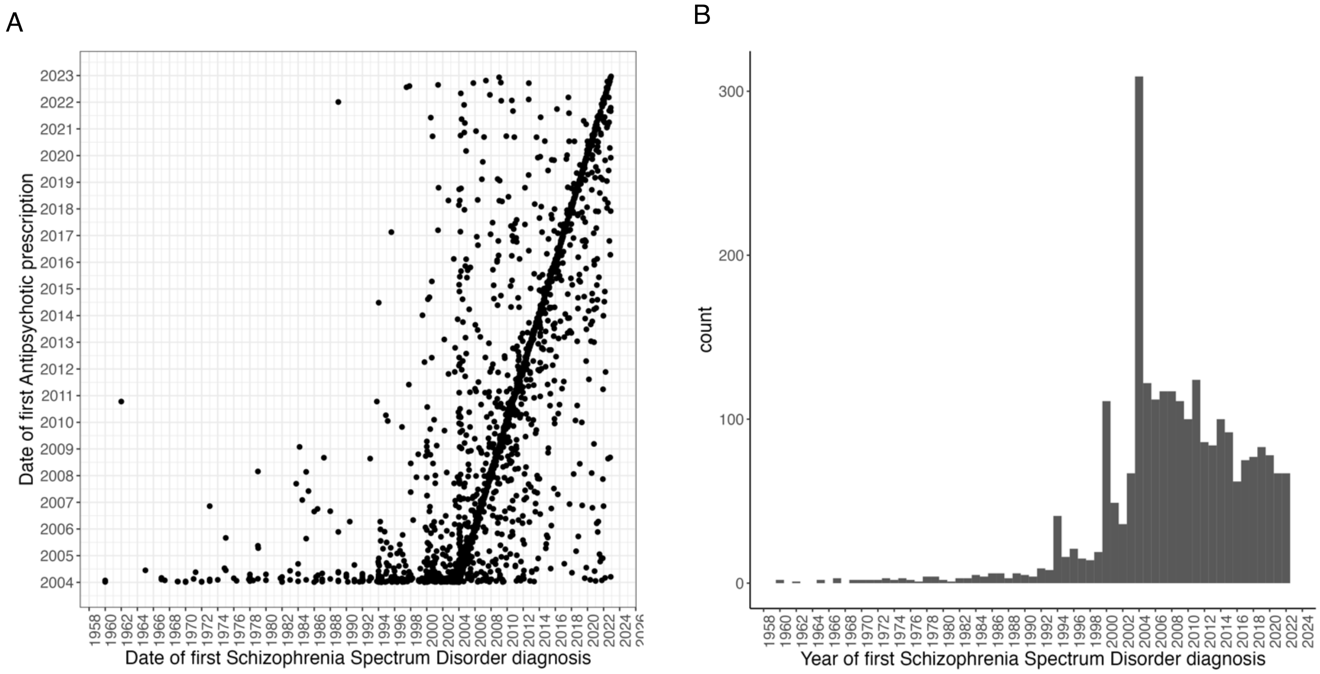

Supplementary Figure 17. Report of the Schizophrenia Spectrum Disorder diagnoses in EstBB. (A) Date of the first Schizophrenia Spectrum Disorder diagnosis vs the date of the first antipsychotic prescription, indicating that many SCZ cases appeared to have received their antipsychotic prescription before their first Schizophrenia Spectrum Disorder diagnosis. (B) Distribution of the years of the first Schizophrenia Spectrum Disorder diagnosis for SCZ cases, highlighting that the majority of SCZ cases received their first diagnosis in 2004 (i.e., when the electronic filing of medical bills started) based on EstBB electronic health records.

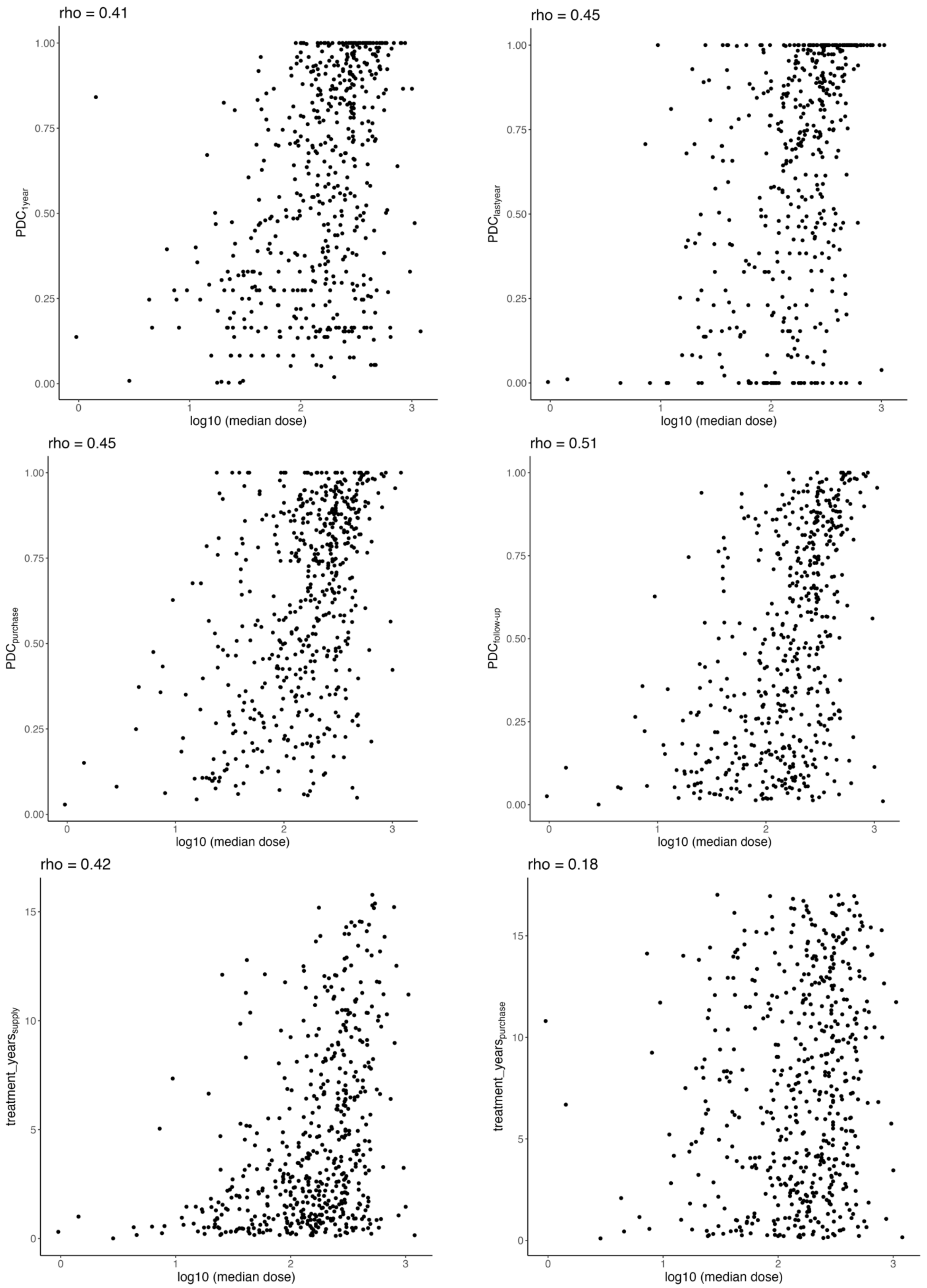

Supplementary Figure 18. Correlations between treatment variables and calculated median chlorpromazine-equivalent dose. Spearman correlations of the calculated median chlorpromazine-equivalent dose values on log10 scale with adherence and treatment length variables.

SUPPLEMENTARY TABLES

Supplementary Table 1. Overview of the availability of smoking, BMI, and genotype data for SCZ cases and controls.

Characteristic	SCZ cases	Controls
Smoking data	625 (92·3%)	2,542 (93·9%)
BMI data	665 (98·2%)	2,693 (99·4%)
Genotype data	577 (85·2%)	2,697 (99·6%)

Supplementary Table 2. Overview of the purchased antipsychotics by SCZ cases (n=595) in EstBB data.

Antipsychotic	Administration Route	Number Of Purchases	Number Of Individuals
Quetiapine	Oral	6168	381
Olanzapine	Oral	4560	320
Aripiprazole	Oral	3452	258
Clozapine	Oral	2014	82
Risperidone	Oral, long-acting injections	1746, 3	204, 1
Haloperidol	Oral, long-acting injections	718, 195	92, 28
Amisulpride	Oral	559	33
Sertindole	Oral	453	44
Flupentixol	Oral, long-acting injections	160, 262	46, 22
Perphenazine	Oral, long-acting injections	202, 80	20, 8
Ziprasidone	Oral	280	18
Levomepromazine	Oral	272	48
Zuclopenthixol	Oral, long-acting injections	24, 200	7, 26
Chlorprothixene	Oral	170	38
Sulpiride	Oral	148	36
Cariprazine	Oral	99	21
Melperone	Oral	90	25
Chlorpromazine	Oral, short-acting injections	52, 1	6, 1
Perphenazine, fluphenazine	Long-acting injection procedure	400	18
Risperidone, olanzapine, aripiprazole, paliperidone	Long-acting injection procedure	839	29

Supplementary Table 3. Results of the association testing between individual PRSs and disease status. Odds ratio (OR), 95% confidence interval (CI), p-value (P).

PRS	OR	95% CI	P
SCZ	1.75	1.58 – 1.94	3.58×10^{-27}
BMI	0.88	0.80 – 0.96	0.0054
CRP	0.88	0.81 – 0.97	0.0065
HDL	1.27	1.03 – 1.23	0.0100
nonHDL	0.90	0.82 – 0.98	0.0196
T2D	0.91	0.83 – 0.99	0.0397
DBP	1.10	1.01 – 1.21	0.0429

Supplementary Table 4. Overview of the results of survival analyses with adherence captured within the first treatment year (PDC_{1year}) as a dependent variable.

Endpoint	N*	N of incident endpoints	PDC_{1year} HR (95%CI)	PRS surviving multiple testing	PRSs only nominally significant**
T2D	365	9	NS	None	TC, nonHDL, T2D
Hypercholesterolemia	328	39	NS	None	None
Essential hypertension	301	42	0.70 (0.51 – 0.97)<br>P = 0.030	None	SBP, BMI, FG, TC
CHD	362	11	NS	None	None
CVD	358	13	NS	None	None
Any metabolic disorder	266	65	0.77 (0.60 – 0.99)<br>P = 0.046	None	SBP

*N of SCZ cases considered in endpoint-specific analyses. **PRSs considered independently in the model.

Supplementary Table 5. Overview of the considered linear mixed models and the results for assessing BMI trajectories over time between SCZ cases and controls. PRS for BMI and PCs are scaled to follow a normal distribution with mean of 0 and SD of 1. Coefficients for BMI age correspond to orthogonal polynomial coefficients.

Model 1: BMI ~ poly(BMI age, 4) + sex + smoking + BMI PRS + PC1 … PC10 + BMI PRS : BMI age + (1 + BMI age | subject)
Model 2: BMI ~ disease status * poly(BMI age, 4) + smoking + sex + BMI PRS + PC1 … PC10 + BMI PRS : BMI age + (1 + BMI age | subject)

Fixed effects for model 1	Beta	SE	P
(Intercept)	26.629	0.135	$<2\times10^{-16}$
poly(BMI_age,1)	156.600	7.188	$<2\times10^{-16}$
poly(BMI_age,2)	-18.600	4.532	4.0×10^{-5}
poly(BMI_age,3)	5.301	3.012	0.078
poly(BMI_age,4)	-7.290	2.458	0.003
sex_female	-1.979	0.154	$<2\times10^{-16}$
smoking_never	-0.394	0.154	0.011
BMI_PRS	0.903	0.252	3.5×10^{-3}
PC1	-0.001	0.076	0.998
PC2	-0.146	0.075	0.053
PC3	-0.092	0.077	0.234
PC4	0.162	0.078	0.037
PC5	-0.079	0.079	0.310
PC6	-0.203	0.076	0.007
PC7	0.018	0.076	0.816
PC8	-0.220	0.077	0.004
PC9	0.022	0.078	0.781
PC10	0.006	0.079	0.937
BMI_PRS:BMI_age	0.019	0.008	0.018

Fixed effects for model 2	Beta	SE	P
(Intercept)	26.466	0.141	$<2\times10^{-16}$
status_SCZ	0.910	0.223	4.6×10^{-4}
poly(BMI_age,1)	141.135	7.793	$<2\times10^{-16}$
poly(BMI_age,2)	-16.147	4.928	0.001
poly(BMI_age,3)	7.982	3.298	0.016
poly(BMI_age,4)	-6.638	2.724	0.015
sex_female	-1.985	0.154	$<2\times10^{-16}$
smoking_never	-0.365	0.155	0.019
BMI_PRS	0.887	0.251	4.2×10^{-4}
PC1	-0.006	0.076	0.936
PC2	-0.155	0.075	0.040
PC3	-0.093	0.077	0.226
PC4	0.149	0.078	0.054
PC5	-0.080	0.078	0.304
PC6	-0.204	0.076	0.007
PC7	-0.006	0.076	0.939
PC8	-0.209	0.077	0.007
PC9	0.025	0.078	0.748
PC10	0.007	0.079	0.928
status_SCZ:poly(BMI_age,1)	92.971	19.458	2.0×10^{-6}
status_SCZ:poly(BMI_age,2)	-15.091	12.330	0.221
status_SCZ:poly(BMI_age,3)	-19.854	8.256	0.016
status_SCZ:poly(BMI_age,4)	-5.888	6.409	0.358
BMI_PRS:BMI_age	0.019	0.008	0.013

Supplementary Table 6. Overview of the considered linear mixed models and the results for assessing BMI trajectories over time between SCZ cases and controls while accounting for treatment length. PRS for BMI, and PCs are scaled to follow a normal distribution with mean of 0 and SD of 1. Coefficients for BMI age and treatment years correspond to orthogonal polynomial coefficients.

Model 1: BMI ~ disease status * poly(BMI age, 4) + sex + smoking + BMI PRS + PC1 … PC10 + BMI PRS : BMI age + (1 + BMI age | subject)
Model 2: BMI ~ disease status * poly(BMI age, 4) + poly(treatment_years$_{supply}$, 4) + sex + smoking + BMI PRS + PC1 … PC10 + BMI PRS : BMI age + (1 + BMI age | subject)

Fixed effects for model 1	Beta	SE	P
(Intercept)	26.466	0.141	$<2\times10^{-16}$
poly(BMI_age,1)	141.135	7.793	$<2\times10^{-16}$
poly(BMI_age,2)	-16.147	4.928	0.001
poly(BMI_age,3)	7.982	3.298	0.016
poly(BMI_age,4)	-6.638	2.724	0.015
status_SCZ	0.910	0.223	4.6×10^{-5}
sex_female	-1.985	0.154	$<2\times10^{-16}$
smoking_never	-0.365	0.155	0.019
BMI_PRS	1.541	0.080	$<2\times10^{-16}$
PC1	-0.006	0.076	0.936
PC2	-0.155	0.075	0.040
PC3	-0.093	0.077	0.226
PC4	0.149	0.078	0.054
PC5	-0.087	0.078	0.304
PC6	-0.080	0.076	0.007
PC7	-0.006	0.076	0.939
PC8	-0.209	0.077	0.007
PC9	0.025	0.078	0.748
PC10	0.007	0.079	0.928
status_SCZ:poly(BMI_age,1)	95.971	19.458	2.0×10^{-6}
status_SCZ:poly(BMI_age,2)	-15.091	12.330	0.221
status_SCZ:poly(BMI_age,3)	-19.854	8.256	0.016
status_SCZ:poly(BMI_age,4)	-5.888	6.409	0.358
BMI_PRS:BMI_age	0.160	0.060	0.013

Fixed effects for model 2	Beta	SE	P
(Intercept)	26.631	0.141	$<2\times10^{-16}$
poly(BMI_age,1)	141.023	7.701	$<2\times10^{-16}$
poly(BMI_age,2)	-16.050	4.880	0.001
poly(BMI_age,3)	8.056	3.276	0.014
poly(BMI_age,4)	-6.628	2.708	0.014
status_SCZ	-0.522	0.257	0.042
poly(treatment_years$_{supply}$,1)	79.401	7.137	$<2\times10^{-16}$
poly(treatment_years$_{supply}$,2)	-10.670	3.489	0.002
poly(treatment_years$_{supply}$,3)	11.170	2.614	2.0×10^{-5}
poly(treatment_years$_{supply}$,4)	-4.436	2.117	0.036
sex_female	-1.960	0.153	$<2\times10^{-16}$
smoking_never	-0.322	0.154	0.037
BMI_PRS	0.863	0.248	5.2×10^{-3}
PC1	-0.032	0.075	0.901
PC2	-0.150	0.075	0.045
PC3	-0.084	0.076	0.271
PC4	0.141	0.077	0.066
PC5	-0.070	0.077	0.384
PC6	-0.197	0.075	0.008
PC7	-0.001	0.076	0.998
PC8	-0.215	0.076	0.005
PC9	0.023	0.077	0.768
PC10	0.010	0.078	0.896
status_SCZ:poly(BMI_age,1)	-8.852	21.386	0.679
status_SCZ:poly(BMI_age,2)	-19.398	12.498	0.121
status_SCZ:poly(BMI_age,3)	-27.291	8.260	9.6×10^{-4}
status_SCZ:poly(BMI_age,4)	-10.163	6.396	0.112
BMI_PRS:BMI_age	0.021	0.008	0.008

Supplementary Table 7. Overview of the linear mixed model and the results for assessing the BMI trajectory over time for SCZ cases while accounting for treatment-associated factors. PRS for BMI and PCs are scaled to follow a normal distribution with mean of 0 and SD of 1.

Model: BMI ~ BMI age + sex + log10($median\_dose_{2years}$) + PDC_{2years} + $treatment\_years_{supply}$ + BMI PRS + smoking + PC1 … PC10 + (1 + BMI age | subject)

Fixed effects	Beta	SE	P
(Intercept)	17.662	1.757	$<2\times10^{-16}$
log10($median\_dose_{2years}$)	1.178	0.379	0.0020
BMI_age	0.177	0.047	0.0002
PDC_{2years}	1.400	0.531	0.0086
$treatment\_years_{supply}$	0.274	0.081	0.0007
sex_female	-1.019	0.640	0.1122
BMI_PRS	1.649	0.305	1.62×10^{-7}
smoking_never	-0.929	0.663	0.1624
PC1	0.280	0.332	0.3991
PC2	-0.231	0.322	0.4747
PC3	-0.468	0.360	0.1951
PC4	-0.172	0.341	0.6147
PC5	-0.014	0.402	0.9720
PC6	-0.261	0.358	0.4668
PC7	0.258	0.330	0.4356
PC8	-0.301	0.308	0.3294
PC9	-0.149	0.303	0.6218
PC10	-0.482	0.307	0.1180
